# ER-mitochondria association negatively affects wound healing by regulating NLRP3 activation

**DOI:** 10.1038/s41419-024-06765-9

**Published:** 2024-06-11

**Authors:** Caterina Licini, Gianluca Morroni, Guendalina Lucarini, Veronica Angela Maria Vitto, Fiorenza Orlando, Sonia Missiroli, Gloria D’Achille, Mariasole Perrone, Tatiana Spadoni, Laura Graciotti, Giorgia Bigossi, Mauro Provinciali, Annamaria Offidani, Monica Mattioli-Belmonte, Oscar Cirioni, Paolo Pinton, Oriana Simonetti, Saverio Marchi

**Affiliations:** 1 Department of Clinical and Molecular Sciences, Marche Polytechnic University, Ancona, Italy; 2 Microbiology Unit, Department of Biomedical Sciences and Public Health, Marche Polytechnic University, Ancona, Italy; 3 Department of Medical Sciences, Laboratory for Technologies of Advanced Therapies (LTTA), University of Ferrara, Ferrara, Italy; 4 Experimental Animal Models for Aging Research, Scientific Technological Area, IRCCS INRCA, 60121 Ancona, Italy; 5 Department of Biomedical Sciences and Public Health, Marche Polytechnic University, Ancona, Italy; 6 Advanced Technology Center for Aging Research, IRCCS INRCA, 60121 Ancona, Italy; 7 Clinic of Dermatology, Department of Clinical and Molecular Sciences, Marche Polytechnic University, Ancona, Italy; 8 Clinic of Infectious Diseases, Department of Biomedical Sciences and Public Health, Marche Polytechnic University, Ancona, Italy

**Keywords:** Energy metabolism, Endoplasmic reticulum, Mechanisms of disease

## Abstract

Methicillin-resistant *Staphylococcus aureus* (MRSA) is the most common causative agent of acute bacterial skin and skin-structure infections (ABSSSI), one of the major challenges to the health system worldwide. Although the use of antibiotics as the first line of intervention for MRSA-infected wounds is recommended, important side effects could occur, including cytotoxicity or immune dysregulation, thus affecting the repair process. Here, we show that the oxazolidinone antibiotic linezolid (LZD) impairs wound healing by aberrantly increasing interleukin 1 β (IL-1β) production in keratinocytes. Mechanistically, LZD triggers a reactive oxygen species (ROS)-independent mitochondrial damage that culminates in increased tethering between the endoplasmic reticulum (ER) and mitochondria, which in turn activates the NLR family pyrin domain-containing 3 (NLRP3) inflammasome complex by promoting its assembly to the mitochondrial surface. Downregulation of ER-mitochondria contact formation is sufficient to inhibit the LZD-driven NLRP3 inflammasome activation and IL-1β production, restoring wound closure. These results identify the ER-mitochondria association as a key factor for NLRP3 activation and reveal a new mechanism in the regulation of the wound healing process that might be clinically relevant.

## Introduction

The acute bacterial skin and skin structure infections (ABSSSIs) definition was introduced by the FDA in 2013 and includes cellulitis/erysipelas, wound infection, and major cutaneous abscess with a minimum lesion surface area of 75 cm^2^ (FDA guidance document https://www.fda.gov/regulatory-information/search-fda-guidance-documents/acute-bacterial-skin-and-skin-structure-infections-developing-drugs-treatment). ABSSSIs are very common infections diffused both in community and hospital settings and mainly sustained by *Staphylococcus aureus* [1]. These species, and in particular the methicillin-resistant *S. aureus* (MRSA) strains, constitute a global burden on healthcare systems: in the USA, the rates of hospitalization due to *S. aureus* skin and soft tissue infections increased dramatically and reached 51% of all *S. aureus* hospitalizations with an incidence of 117 cases per 100,000 populations [2].

The first-line therapy for the management of ABSSSI due to MRSA is vancomycin [3], a first-generation glycopeptide with broad activity against Gram-positive bacteria, but alternative therapies included many options that demonstrated non-inferiority to glycopeptides, such as ceftaroline, linezolid, dalbavancin, or daptomycin [4]. Although the use of antibiotics and antibacterial products for the management of MRSA-infected wounds is mandatory, considerable doubts still exist regarding the efficacy of antibiotics in accelerating wound repair, mainly due to their putative detrimental effects on both fibroblasts and keratinocytes [5–7]. These cells, particularly keratinocytes, are deeply involved in the stage of re-epithelization, one of the major phases of the healing process and universally used as a distinctive parameter of successful wound closure [8].

Normal closure and tissue repair consist of keratinocyte differentiation, migration, and proliferation at the edge of the wound, to extend the newly formed epithelial carpet made of several layers of cells in the epidermis [9]. Importantly, keratinocytes actively contribute to innate immunity during skin repair, producing a series of cytokines, chemokines, and antimicrobial peptides that virtually regulate all phases of healing [10–12]. In this context, keratinocytes initiate and boost the immune response, but also show mechanisms to restrict and minimize inflammation, which is crucial for the success of the healing process [13]. Therefore, a persistent inflammatory phase is a typical trait of non-healing (chronic) wounds, which are characterized by undifferentiated keratinocytes undergoing divisions throughout the suprabasal layers, thereby composing a hyper-proliferative epidermis (hypertrophic epithelium) [14, 15]. However, whether antibiotics might affect wound closure by altering specific intracellular mechanisms and the immune functions of keratinocytes, is still obscure.

Here, we focused on linezolid (LZD), the first antibiotic of the synthetic class of oxazolidinones, as a means to interrogate the effects of antibiotic-mediated damage on wound repair. Since its approval in the 2000s, LZD represents a last resort option to treat severe infections sustained by Gram-positive bacteria and in particular MRSA, vancomycin-resistant enterococci (VRE), and multi-drug resistant streptococci [16]. Despite its favorable pharmacodynamic-pharmacokinetic profiles and the low rates of resistance, the prolonged courses with LZD are associated with some side effects such as diarrhea, headache, nausea, vomiting, and reversible myelosuppression (mostly thrombocytopenia) [17]. These adverse events seem to be related to the mechanisms of action of the oxazolidinone class, which targets the peptidyl-transferase center of the bacterial ribosome but also interferes with the mitochondrial activity and protein synthesis by blocking mitochondrial ribosomes [18–20]. Mitochondria play an essential role in the control of inflammation [21], and different mitochondrial aberrations have been associated with multiple disorders encompassing deregulated inflammatory response [22]. The consequence of interfering with mitochondrial functions in keratinocytes and wound healing process, however, has not been well elucidated.

In this study, we show that LZD treatment of MRSA-infected wounds significantly delays skin repair, mainly due to an excess of interleukin 1 β (IL-1β) in keratinocytes. Mechanistically, LZD triggers mitochondrial damage that culminates in increased tethering between the endoplasmic reticulum (ER) and mitochondria, which in turn activates the NLR family pyrin domain-containing 3 (NLRP3) inflammasome. By downregulating the ER-mitochondria connection, we inhibit the LZD-driven NLRP3 inflammasome activation and IL-1β production, restoring wound closure.

## Results

### LZD impairs wound healing in vivo

**Fig. 1:**
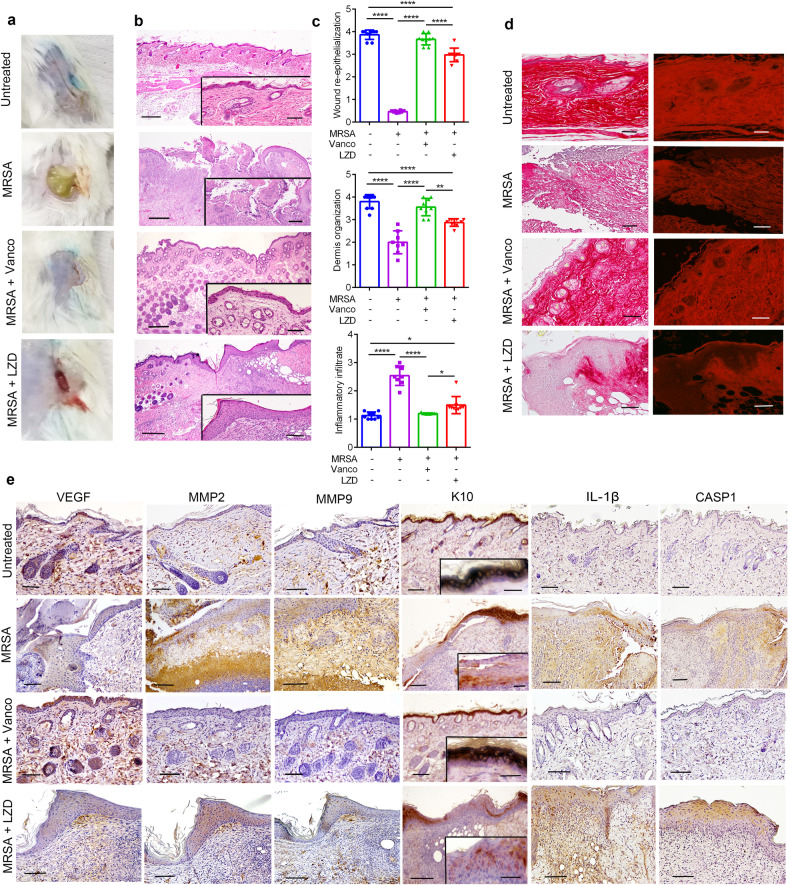
LZD inhibits healing in a murine model of MRSA-infected wound. **a** Representative images of wound closure in mice, treated as indicated, at day 10. **b** Representative histology of the wound healing in mice, treated as indicated, at day 10 (skin sections; H&E staining). Scale bars; 100 µm; inserts: 40 µm. **c** Evaluation of wound healing by different histological parameters (One-way ANOVA: *P = 0.0109 [Inflammatory infiltrate: uninfected/untreated vs MRSA/LZD]; *P = 0.0497 [Inflammatory infiltrate: MRSA/Vanco vs MRSA/LZD]; **P = 0.0018; ****P < 0.0001). **d** Representative Sirius Red staining of collagen organization in wounded skin sections of mice, treated as indicated, at day 10 (scale bars: 40 µm). Fluorescent images on the right. **e** Representative immunohistochemistry staining images of different wound healing markers, taken from skin sections of mice, treated as indicated. Scale bars: 40 µm; inserts: 20 µm.

To test the effects of LZD on wound healing, we used a mouse model of wound infected with MRSA [23]. Macroscopic observation on day 10 (Fig. 1a) revealed deposition of pus and large wound area in the MRSA-infected mice. Conversely, the epithelial tongues converged to completely cover both the uninfected wounds and those treated with vancomycin, whereas in the LZD-treated wounds, the epithelial gap was still evident, covered with a thick scab (Fig. 1a). Histological evaluation, according to specific wound healing criteria (Supplementary Table 1), showed typical traits of compromised repair in MRSA-infected animals, including incomplete central re-epithelialization, a markedly thicker epithelium in the edges, abundant inflammatory infiltrate, and a poor organization of the dermis (Fig. 1b, c). The vancomycin-treated group displayed a robust epidermal coverage, with reconstitution of the regular and keratinized epidermal lining, and few inflammatory cells, whereas LZD significantly delayed the wound repair, as evidenced by pronounced hypertrophy of the epithelium, a modest dermis organization, and a marked increase in inflammatory cells infiltration (Fig. 1b, c). Analysis of the collagen network through Sirius Red staining confirmed the impaired wound healing in LZD-treated group, which exhibited a disorganized structure and decreased deposition of collagen fibers compared to vancomycin (Fig. 1d and Supplementary Fig. 1a). All these effects cannot be ascribed to inadequate bacterial clearance, since LZD displayed a superior antibacterial activity than vancomycin (Supplementary Fig. 1b), indicating that LZD affects wound healing by targeting specific cellular events.

To investigate the LZD-induced defects in wound repair at the molecular level, we analyzed different markers of healing by immunohistochemistry. Compared to uninfected and vancomycin-treated groups, LZD reduced the levels of vascular endothelial growth factor (VEGF) and aberrantly increased matrix metalloproteinases (MMPs), especially MMP2 (Fig. 1e and Supplementary Table 2), reflecting a condition of impaired wound closure [24]. Accordingly, the expression of the keratinocyte differentiation marker keratin 10 (K10) in the uppermost layers of the wound bed, the neo-epidermis, was absent in MRSA group and only focally stained in LZD-treated mice (Fig. 1e and Supplementary Table 2). LZD promotes IL-1β production [25], and persistent IL-1β-mediated inflammatory signals in wounds evoke a cascade of events that drastically impinges on repair and regeneration [26, 27]. Thus, we examined if high levels of IL-1β could be associated with the impaired healing process observed upon LZD addiction. IL-1β is virtually undetectable in both uninfected and vancomycin-treated wounds, whereas it was highly expressed in LZD-treated mice, prevalently in the epithelium and infiltration tissue (Fig. 1e and Supplementary Table 3). These data were supported by analysis of caspase 1 (CASP1) immunoreactivity, which was robust in both MRSA and LZD groups, especially in the epithelial cells, whereas it was negligible in the other conditions (Fig. 1e and Supplementary Table 3).

Taken together, these findings indicated that despite its high efficiency as antibacterial agent, LZD severely impairs the normal wound healing response, which coincides with the aberrant upregulation of IL-1β levels.

### LZD stimulates IL-1β secretion in keratinocytes by promoting NLRP3 docking to mitochondria

**Fig. 2:**
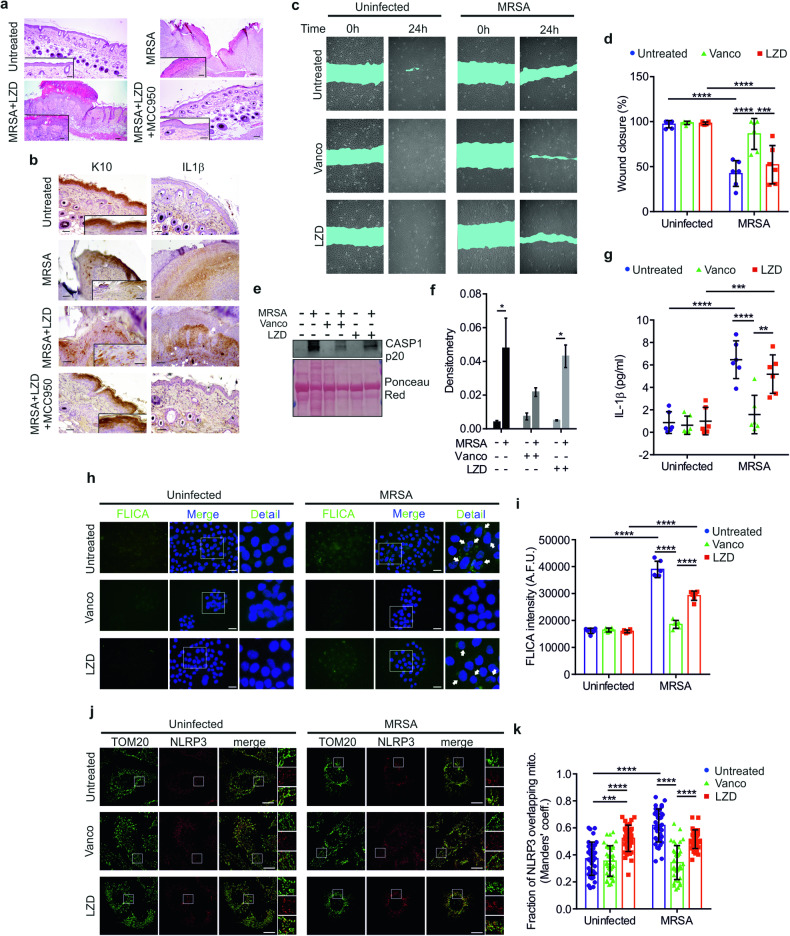
LZD promotes IL-1β secretion via NLRP3 activation. **a** Representative histology of the wound healing in mice, treated as indicated, at day 10 (skin sections; H&E staining). Scale bars; 100 µm; inserts: 40 µm. **b** Representative immunohistochemistry staining images of wounded skin sections, taken from mice, treated as indicated. Scale bars: 40 µm; inserts: 20 µm. **c** Representative images at times 0 and 24 h from wound opening in HaCaT cells, treated as indicated. **d** HaCaT cells were treated as indicated. Quantification of wound closure (showed as percentage) has been performed 24 h after wound opening (*n* = 3 independent experiments; Two-way ANOVA: ***P = 0.0008; ****P < 0.0001). **e** Representative immunoblot for Caspase 1 p20 secreted into the culture medium of HaCaT cells, treated as indicated. **f** Densitometric analysis of immunoblots for Caspase p20 in HaCaT culture media, normalized on Ponceau Red staining (*n* = 3 independent experiments; Two-way ANOVA: *P = 0.0197 [uninfected/untreated vs MRSA/untreated]; *P = 0.0472 [uninfected/LZD vs MRSA/LZD]). **g** Quantification of IL-1β secreted into supernatants from HaCaT cells, treated as indicated, performed by ELISA assay (*n* = 3 independent experiments; Two-way ANOVA: **P = 0.0015; ***P = 0.0002; ****P < 0.0001). **h** Representative images of Caspase 1 activation (green) in HaCaT cells treated as indicated, detected by FAM-FLICA (scale bars: 10 μm). Magnifications of Caspase 1 activation (white arrows) have been reported. **i** Quantification of the FLICA intensity in HaCaT cells, treated as indicated (*n* = 3 independent experiments; Two-way ANOVA: ****P < 0.0001). **j** Representative confocal immunofluorescence staining images of NLRP3 (red) and TOM20 (used as a mitochondrial marker, green) in HaCaT cells, treated as indicated (scale bars: 5 μm). Merged images are shown. Magnification of TOM20, NLRP3, and merged images in insets. **k** Quantification of NLRP3/mitochondria association in HaCaT cells, treated as indicated, by Manders’ coefficient calculation (*n* = 3 independent experiments; Two-way ANOVA: ***P = 0.0003; ****P < 0.0001).

The proteolytic maturation of IL-1β mainly entails the recruitment/activation of CASP1 to the NLRP3 inflammasome, a multiprotein complex that also includes the sensing subunit NLRP3 and the adapter protein PYD and CARD domain containing (PYCARD; best known as ASC) [28]. The activation of NLRP3 inflammasome consists of a two-step process: a first “priming” event, which mainly serves to upregulate the expression of inflammasome components, and a second step to fully activate the complex [29]. LZD has been described as a potential NLRP3 activator in macrophages [25]. Thus, we investigated whether pharmacological inhibition of NLRP3 could restore healing in LZD-treated wounds. As shown in Fig. 2a, addiction of the NLRP3 inhibitor MCC950 ameliorated wound repair in the MRSA-infected/LZD-treated group, evidenced by increased skin differentiation with development of new hair follicles, reduced inflammatory infiltrate, and well-assembled collagen matrix in the skin dermis. These observations were supported by analysis of K10 immunoreactivity, showing abundant K10-positive cells located in suprabasal and uppermost layers at the wound edges and center level, after MCC950 treatment (Fig. 2b). Moreover, IL-1β immunopositive cells were not detected (Fig. 2b), confirming that the LZD-induced IL-1β production was mainly ascribed to NLRP3 activation.

Human keratinocytes contain all the key components of the inflammasome [30] and release IL-1β in response to many stimuli, including *S. aureus* [31]. MRSA infection of HaCaT cells for 6 h was sufficient to induce CASP1 autoproteolytic activation (Supplementary Fig. 2a), in agreement with previous results [32]. The MRSA-dependent CASP1 processing was not secondary to putative MRSA-induced cytotoxicity, since we observed multiple mitotic events during the first hours of infection (Supplementary Video 1) and only a slight reduction in cell viability at longer times (Supplementary Fig. 2b). Analysis of keratinocytes migration using a wound healing scratch assay revealed that vancomycin mostly abolished the detrimental effects driven by MRSA, whereas LZD did not (Fig. 2c, d). Importantly, both MCC950 and the IL-1 receptor antagonist Anakinra were able to improve wound closure in MRSA-infected/LZD-treated cells (Supplementary Fig. 2c, d), indicating IL-1β overproduction as the key event in the inhibition of wound repair induced by LZD. The involvement of pyroptotic cell death couples with IL-1β secretion has been excluded, since silencing of the pyroptosis executor gasdermin D (GSDMD) (Supplementary Fig. 2e) did not affect wound closure (Supplementary Fig. 2f). Moreover, MRSA was able to induce IL-1β release also in *GSDMD*-silenced cells (Supplementary Fig. 2g), indicating that, in our conditions, IL-1β secretion could occur in a GSDMD-independent manner [33].

LZD induced the activation of CASP1 (Fig. 2e, f) and consequent IL-1β secretion (Fig. 2g) in HaCaT cells, as well as in primary normal Human Epidermal Keratinocytes (nHEK) (Supplementary Fig. 2h), indicating a non-cell autonomous activity. Of note, LZD alone was unable to generate mature IL-1β, reasonably due to the lack of priming stimuli. Accordingly, we detected LZD-mediated CASP1 activation in intact keratinocytes only upon infection with MRSA (Fig. 2h, i), which implies the formation of active inflammasome platforms.

Mitochondria play a crucial role in the coordination of the inflammatory response [21] and NLRP3 associates with the mitochondrial surface to ensure the complete activation of the whole complex [34]. Therefore, we examined if LZD could positively regulate NLRP3 activity by favoring its localization to mitochondria. We observed an augmented fraction of NLRP3 overlapping mitochondria in MRSA-infected and MRSA-infected/LZD-treated cells, as well as with LZD alone, whereas vancomycin had no effects (Fig. 2j, k). Similar data have been obtained in nHEK (Supplementary Fig. 2I, j). Moreover, biochemical isolation of mitochondria showed that ASC, like NLRP3, translocated to mitochondria upon LZD treatment or with MRSA alone (Supplementary Fig. 2k).

Overall, these results demonstrated that the LZD-mediated impairment of wound closure correlates with the recruitment and activation of the NLRP3 inflammasome to the mitochondrial compartment.

### LZD induces mitochondrial damage

**Fig. 3:**
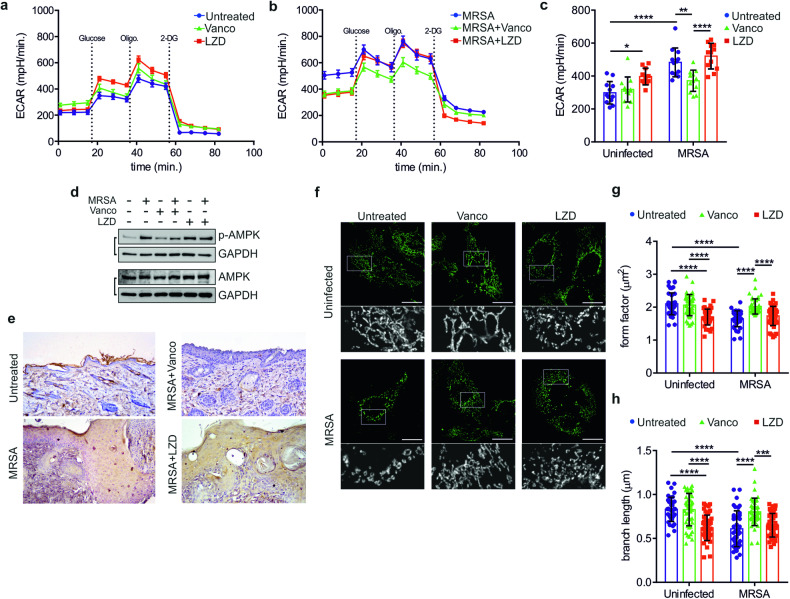
LZD induces mitochondrial damage. **a** Evaluation of glycolytic activity by the extracellular acidification rate (ECAR) in uninfected HaCaT cells, with or without addiction of Vancomycin or LZD. The additions of glucose, oligomycin (Oligo.), and deoxyglucose (2-DG) are indicated as dotted, vertical lines. **b** Evaluation of glycolytic activity by the extracellular acidification rate (ECAR) in MRSA-infected HaCaT cells, with or without addiction of Vancomycin or LZD. The additions of glucose, oligomycin (Oligo.), and deoxyglucose (2-DG) are indicated as dotted, vertical lines. **c** Quantification of glycolysis in HaCaT cells, treated as indicated (*n* = 3 independent experiments; Two-way ANOVA: *P = 0.0193; **P = 0.0042; ****P < 0.0001). **d** Representative immunoblot for AMPK and phosphorylated AMPK (p-AMPK; Thr172) in HaCaT cells, treated as indicated **e** Representative immunohistochemistry staining of p-AMPK in wounded skin sections, taken from mice treated as indicated. **f** Representative confocal images of mitochondrial morphology (TOM20) in HaCaT cells, treated as indicated (scale bars: 5 µm). Magnifications in insets. **g** Morphometric analysis of the mitochondrial network in HaCaT cells, treated as indicated, by calculation of the parameter “form factor” (*n* = 3 independent experiments; Two-way ANOVA: ****P < 0.0001). **h** Analysis of mitochondrial network connectivity in HaCaT cells, treated as indicated, by calculation of the parameter “branch length” (*n* = 3 independent experiments; Two-way ANOVA: ***P = 0.0002; ****P < 0.0001).

Numerous danger signals triggering NLRP3 activation involve different molecular pathways that in turn emerge from mitochondrial dysfunctions [35, 36]. LZD usage is associated with mitochondrial toxicity [37]. Thus, we aimed to explore the mitochondrial alterations evoked by LZD in keratinocytes. Firstly, we assessed that LZD inhibited respiratory complex IV (C-IV) activity (Supplementary Fig. 3a, b), as previously suggested [20]. As expected, LZD markedly reduced mitochondrial respiration (Supplementary Fig. 3c), which coincided with parallel stimulation of aerobic glycolysis (Fig. 3a). Moreover, vancomycin showed no side effects on mitochondrial bioenergetics (Supplementary Fig. 3d), and its antibacterial activity is sufficient to mitigate the activation of glycolysis induced by MRSA (Fig. 3a–c). Consistent with the metabolic data, the energy sensor AMP-activated protein kinase (AMPK) [38] was activated in response to those stimuli that are capable to initiate glycolysis, both in vitro (Fig. 3d and Supplementary 3e) and in vivo, where the expression of phosphorylated AMPK was robust at the epithelial and dermal level in mice infected with MRSA alone or upon LZD addiction, while it was absent in uninfected and vancomycin-treated wounds (Fig. 3e). Next, we analyzed if the metabolic stress elicited by LZD would coexist with morphological defects of mitochondria. Both LZD, either in non-infected and infected cells, and MRSA evoked a drastic remodelling of the mitochondrial network, evidenced by accumulation of spherical and toroidal mitochondria, as well as reduction of mitochondrial interconnections, whereas vancomycin inhibited extensive fragmentation (Fig. 3f–h). The mitochondrial damage induced by MRSA also involved the dissipation of mitochondrial transmembrane potential (Supplementary Fig. 3f), as described for multiple bacterial pathogens that target mitochondria [39]. Conversely, LZD-treated cells displayed normal electrochemical gradient despite the electron transport chain imbalance, similar to mtDNA-depleted (ρ^0^) cells [40].

### LZD increases ER-mitochondria contacts

**Fig. 4:**
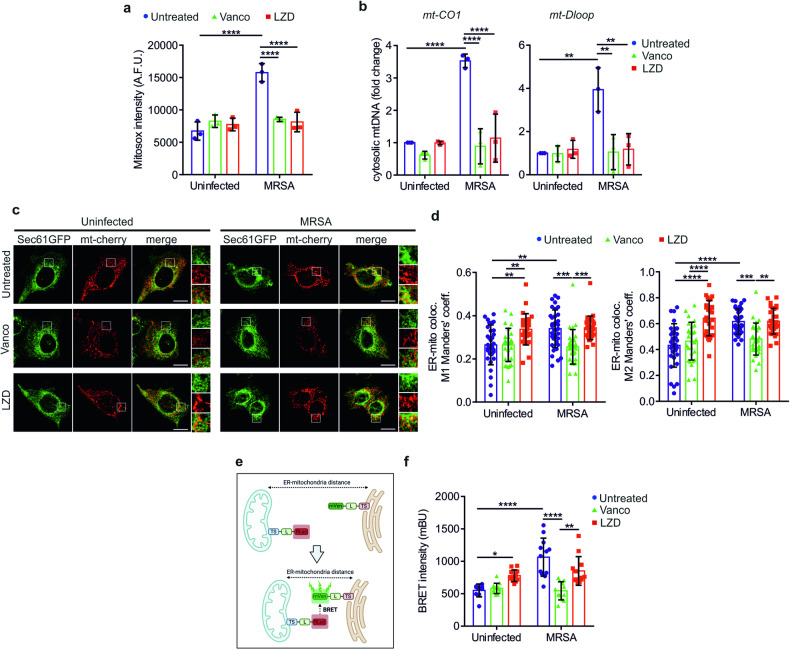
LZD increases ER-mitochondria association. **a** Mitochondrial Superoxide levels in HaCaT cells, treated as indicated, were detected by Mitosox probe (*n* = 3 independent experiments; Two-way ANOVA: ****P < 0.0001). **b** Cytosolic mtDNA evaluation by the analysis of *mt-CO1* and *mt-Dloop* levels, in HaCaT cells, treated as indicated (*n* = 3 independent experiments; Two-way ANOVA: **P = 0.0014 [uninfected/untreated vs MRSA/untreated]; **P = 0.0016 [MRSA/untreated vs MRSA/Vanco]; **P = 0.0023 [MRSA/untreated vs MRSA/LZD]; **** *P* < 0.0001). **c** Representative confocal images of ER-mitochondria association in HaCaT cells, transiently transfected with SEC61-GFP (ER marker) and mitochondrial-targeted cherry (mt-cherry, mitochondrial marker), and then treated as indicated (scale bars: 5 µm). Merged images are shown. Magnification of SEC61-GFP, mt-cherry, and merged images in insets. **d** Quantification of ER-mitochondria associations by Manders’ coefficients calculation in HaCaT cells, treated as indicated (*n* = 3 independent experiments; Two-way ANOVA: **P = 0.0024 [M1: uninfected/untreated vs MRSA/untreated]; **P = 0.0080 [M1: uninfected/untreated vs uninfected/LZD]; **P = 0.0092 [M1: uninfected/Vanco vs uninfected/LZD]; ***P = 0.0005 [M1: MRSA/untreated vs MRSA/Vanco]; ***P = 0.0005 [M1: MRSA/Vanco vs MRSA/LZD]; ***P = 0.0009 [M2: MRSA/untreated vs MRSA/Vanco]; **P = 0.0012 [M2: MRSA/Vanco vs MRSA/LZD]; ****P < 0.0001). **e** Schematic drawing representing principles of the BRET method. Bioluminescence is developed as a consequence of the proximity of R-Luc (mitochondria) and mVenus (ER). **f** Quantification of bioluminescence intensity detected by BRET assay, in HaCaT cells, treated as indicated (*n* = 3 independent experiments; Two-way ANOVA: *P = 0.0383; ***P = 0.0008; ****P < 0.0001).

To correlate these mitochondrial dysfunctions with NLRP3 regulation, we investigated the main mitochondrial damage-associated molecular patterns (mtDAMPs) that have been proposed as NLRP3 activators, including reactive oxygen species (ROS) and cytosolic mtDNA [21]. Both vancomycin and LZD abolished the MRSA-mediated mitochondrial ROS overproduction (Fig. 4a), suggesting that LZD could activate NLRP3 in a ROS-independent manner. Furthermore, LZD did not promote mtDNA release into the cytoplasm, as evidenced by RT-PCR (Fig. 4b) and live imaging (Supplementary Fig. 4a, b). We thereby searched for alternative mitochondrial mechanisms underlying NLRP3 activation by LZD. Mitochondrial Ca^2+^ entry through the mitochondrial calcium uniporter (MCU) complex has been indicated as a positive regulator of NLRP3 inflammasome [41, 42]. However, LZD did not affect mitochondrial Ca^2+^ uptake, either alone or in combination with MRSA (Supplementary Fig. 4c, d). Mitochondria juxtapose with the ER membranes to form structural and functional connections, known as ER-mitochondria contact sites, which control multiple cellular processes and could vary in both length and number upon different stimuli, including stressful conditions [43, 44]. During their activation process, NLRP3 and ASC translocate, at least transitorily, at the ER-mitochondria interface [45, 46]. Nevertheless, the spatial rearrangement of intracellular organelles appeared crucial for NLRP3 trafficking and optimal inflammasome activation [47–50]. Confocal pseudo co-localization revealed that both LZD and MRSA were capable to increase ER-mitochondria contacts in live cells, whereas vancomycin had no effects (Fig. 4c, d). Moreover, using a bioluminescence resonance energy transfer (BRET)-based approach (Fig. 4e) [51], we validated that LZD, alone or in MRSA-infected cells, enhanced the ER-mitochondria association (Fig. 4f). Notably, MCC950 did not alleviate the LZD-mediated metabolic defects (Supplementary Fig. 4e), morphological alterations (Supplementary Fig. 4f), and aberrant ER-mitochondria coupling (Supplementary Fig. 4g), suggesting that NLRP3 activation is a consequence of the mitochondrial damage induced by LZD.

These findings mainly indicated that i) LZD activated NLRP3 inflammasome independently from ROS and mtDNA leakage, and ii) the mitochondrial derangements induced by LZD, as well as MRSA, culminated in increased ER-mitochondria tethering.

### Downregulation of ER-mitochondria association inhibits LZD-mediated NLRP3 inflammasome activation

**Fig. 5:**
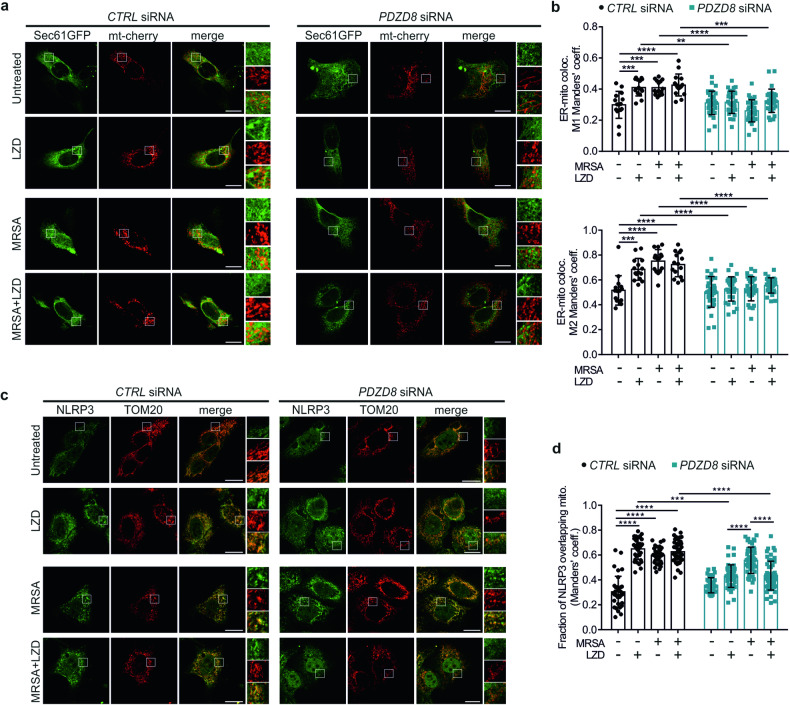
LZD-mediated NLRP3 activation depends on ER-mitochondria contact increase. **a** Representative confocal images of ER-mitochondria association in control (negative siRNA) and *PDZD8*-silenced HaCaT cells, transiently transfected with SEC61-GFP (ER marker) and mitochondrial-targeted cherry (mt-cherry, mitochondrial marker), and then treated as indicated (scale bars: 5 µm). Merged images are shown. Magnification of SEC61-GFP, mt-cherry, and merged images in insets. **b** Quantification of ER-mitochondria associations by Manders’ coefficients calculation in control (negative siRNA) and *PDZD8*-silenced HaCaT cells, treated as indicated (*n* = 3 independent experiments; Two-way ANOVA: **P = 0.0011 [M1: CTRL siRNA uninfected/LZD vs PDZD8 sirna uninfected/LZD]; ***P = 0.0008 [M1: CTRL siRNA uninfected/untreated vs CTRL siRNA uninfected/LZD]; ***P = 0.0010 [M1: CTRL siRNA uninfected/untreated vs CTRL siRNA MRSA/untreated]; ***P = 0.0004 [M1: CTRL siRNA MRSA/LZD vs PDZD8 siRNA MRSA/LZD]; ***P = 0.0001 [M2: CTRL siRNA uninfected/untreated vs CTRL siRNA uninfected/LZD]; ****P < 0.0001). **c** Representative confocal immunofluorescence staining images of NLRP3 (green) and TOM20 (used as a mitochondrial marker, red) in control (negative siRNA) and *PDZD8*-silenced HaCaT cells, treated as indicated (scale bars: 5 µm). Merged images are shown. Magnification of TOM20, NLRP3, and merged images in insets. **d** Quantification of NLRP3/mitochondria association in control (negative siRNA) and *PDZD8*-silenced HaCaT cells, treated as indicated, by Manders’ coefficient calculation (*n* = 3 independent experiments; Two-way ANOVA: ***P = 0.0008; ****P < 0.0001.

Although several molecular events governing the inflammatory signaling, including antiviral or antibacterial pathways, originate at both ER and mitochondria [52], if changes in the physical association between the two organelles affect the immune response is still elusive [53, 54]. To understand the real contribution of ER-mitochondria contacts in the process of NLRP3 inflammasome activation, we targeted the PDZ domain-containing protein 8 (*PDZD8*) gene, which encodes for a crucial component of an ER-mitochondria tether [55–57]. Downregulation of *PDZD8* (Supplementary Fig. 5a) abolished the increase of ER-mitochondria interactions induced by both LZD and MRSA, measured either by confocal co-localization (Fig. 5a, b) or BRET technique (Supplementary Fig. 5b).

The enhanced ER-mitochondria proximity observed in LZD-treated and MRSA-infected cells reflected the ability of NLRP3 to dock at mitochondria (Fig. 2k, l and Supplementary 2e, f). To test this correlation, we measured the amount of NLRP3 overlapping mitochondria in *PDZD8*-depleted keratinocytes. Unexpectedly, *PDZD8* silencing diminished the quote of mitochondrial NLRP3 only upon LZD treatment (alone or in combination with MRSA), without affecting NLRP3 translocation in MRSA-infected cells (Fig. 5c, d). Of note, the other mitochondrial alterations elicited by LZD, such as metabolic and morphological remodelling, were not influenced by PDZD8 downregulation (Supplementary Fig. 5c, d), which indicates that the lower NLRP3 activity was primarily due to reduction in ER-mitochondria proximities.

### Blocking ER-mitochondria contact formation restores healing in LZD-treated wounds

**Fig. 6:**
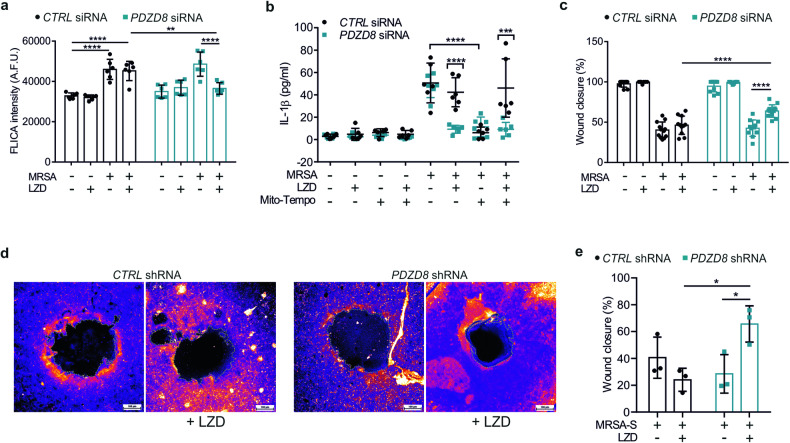
Downregulation of ER-mitochondria tethering improves closure of LZD-treated wounds. **a** Quantification of the FLICA intensity in control (negative siRNA) and *PDZD8*-silenced HaCaT cells, treated as indicated (*n* = 3 independent experiments; Two-way ANOVA; **P = 0.0081; ****P < 0.0001). **b** Quantification of IL-1β secreted into supernatants from control (negative siRNA) and *PDZD8*-silenced HaCaT cells, treated as indicated, performed by ELISA assay (*n* = 3 independent experiments; Two-way ANOVA: ***P = 0.0004; ****P < 0.0001). **c** Control (negative siRNA) and *PDZD8*-silenced HaCaT cells were treated as indicated. Quantification of wound closure (showed as percentage) has been performed 24 h after wound opening (*n* = 3 independent experiments; Two-way ANOVA: ****P < 0.0001). **d** Wound healing in the 3D-wound model. The colored parts represent pLKO-transfected (*CTRL* shRNA) and *PDZD8* shRNA-transfected stable HaCaT cells. Colors from blue to yellow denote low to higher cell density, respectively. Scale bar: 100 µm. **e** Quantification of wound closure (showed as percentage) in the 3D-wound healing assay (*n* = 3 independent experiments; Two-way ANOVA: *P = 0.0202 [CTRL shRNA MRSA-S/LZD vs PDZD8 shRNA MRSA-S/LZD]; *P = 0.0356 [PDZD8 shRNA MRSA-S vs PDZD8 shRNA MRSA-S/LZD]).

The reduction of ER-mitochondria contacts blocked the activation of CASP1 triggered by LZD, but not MRSA (Fig. 6a), suggesting that the increased tethering is a crucial event in the mechanism of NLRP3 activation induced by LZD. Analysis of IL-1β secretion confirmed that PDZD8 depletion abrogated the pro-inflammatory functions of LZD, whereas MRSA-infected cells were sensitive to treatment with the mitochondria-targeted antioxidant mito-Tempo (Fig. 6b).

Finally, we examined whether reducing the immunogenic effects of LZD through inhibition of ER-mitochondria connection could affect wound repair. The scratch assay showed that knockdown of PDZD8 significantly ameliorated wound closure in LZD-treated keratinocytes (Fig. 6c). Nevertheless, mitochondrial ROS scavenging partially rescued the negative effects produced by MRSA on cell migration (Supplementary Fig. 6a). Encouraged by the above results, we created a 3D model of wound repair consisting of fibroblasts and keratinocytes in a collagen matrix (Supplementary Fig. 6b) and mimicked persistent inflammation by using MRSA supernatant (MRSA-S). Repeated exposure with diluted (20%) MRSA-S did not affect cell viability (Supplementary Fig. 6c) but delayed keratinocyte migration and wound healing in the 2D model (Supplementary Fig. 6d), in accordance with previous results [58]. Thus, we generated stable *PDZD8* knockdown keratinocytes (Supplementary Fig. 6e) and analyzed wound closure in our skin-equivalent system, treated with MRSA-S, with or without addiction of LZD. Figure 6d, e shows that low levels of PDZD8 did not affect the migratory activity of keratinocytes upon treatment with MRSA-S alone, but strongly improved wound closure in response to LZD.

Collectively, these results revealed that blocking the ER-mitochondria contacts increase by downregulation of PDZD8 completely abrogated the LZD-mediated activation of NLRP3 inflammasome and IL-1β production, thus alleviating, at least in part, the detrimental effects of the antibiotic on wound healing.

## Discussion

Our findings revealed three significant aspects of mitochondrial dynamics in controlling skin repair and NLRP3 inflammasome activation. First, LZD showed detrimental effects on the re-epithelization of infected wounds, which are due, at least in part, to excessive IL-1β production. Accordingly, sustained NLRP3 inflammasome activation has been correlated with defective wound repair in diabetic and aged mice [59]. However, inadequate healing has been described in NLRP3-null mice, as a consequence of the suppression of the early inflammatory phase, which is crucial for efficient skin repair, as well as maximal bacterial clearance [60]. Based on these considerations, we designed an in vivo protocol that included the administration of MCC950 only in the late phase (from day 5 to 10) of healing, which provided significant benefits by minimizing the LZD-driven persistent inflammation. Thus, NLRP3 inhibition could mitigate the negative effects of antibiotics on wound closure only by following a well-defined treatment schedule.

Second, MRSA strongly impaired mitochondrial functions using its reservoir of virulent factors, as evidenced by mitochondrial fragmentation, metabolic rewiring, and reduction of membrane potential. Such morpho-functional alterations culminated with mitochondrial ROS generation and mtDNA release, forming oxidized mtDNA that trigger NLRP3 inflammasome activation [61, 62]. These events have been indicated as strategic for the host to counteract *S. aureus* infection [31, 63], pointing out mitochondria as critical players in the orchestration of defence to restrict *S. aureus* dissemination. Contrarily to LZD, the increased ER-mitochondria contacts induced by MRSA appeared dispensable for NLRP3 activation, suggesting that a mitochondrial dysfunction encompassing ROS accumulation and/or mtDNA leakage is sufficient to ensure NLRP3 localization at mitochondria and activation, thereby masking the potential role of ER-mitochondria juxtapositions as positive regulators of the complex. On the other hand, MRSA toxins could also activate other inflammasome platforms and sustain IL-1β production in an NLRP3-independent manner, as previously suggested [32], thus justifying the capacity of MRSA to elicit IL-1β secretion despite PDZD8 downregulation.

Third, we showed that the LZD-driven mitochondrial damage induced a non-canonical, ROS-independent activation of NLRP3 inflammasome, which relied on increased ER-mitochondria proximities. In this context, ER stress can trigger inflammation via activation of NLRP3 [64], as well as promote ER-mitochondria tethering [65]. However, whether the modulation of organelles coupling directly interferes with NLRP3 activation was heretofore unknown. Here, we observed that by targeting PDZD8, an ER transmembrane protein whose depletion drastically reduces ER-mitochondria proximities [55], we inhibited the NLRP3 translocation to the mitochondrial surface and IL-1β production elicited by LZD, but not MRSA, demonstrating that ER-mitochondria contacts are essential to provide NLRP3 activation under specific conditions, and specifically in a ROS-independent fashion. Of note, the ER-mitochondria tethering factor mitofusin 2 (MFN2) [66] has been proposed as a critical regulator of NLRP3 activation upon RNA virus infection [67]. Accordingly, the E3 ubiquitin-protein ligase membrane-associated ring-CH-type finger 5 (MARCHF5, also known as MARCH5 or MITOL), which positively regulates ER-mitochondria contact formation [68], has been implicated in NLRP3 inflammasome assembly and activation at the mitochondria in a recent preprint (not peer-reviewed) [69].

The capacity of LZD to stimulate IL-1β production via NLRP3 has been associated with the exposure of the phospholipid cardiolipin to the outer mitochondrial membrane [25], which is another potential element into NLRP3 inflammasome activation. Our work does not address or rebut this mechanistic route. However, it is important to note that experimental approaches aimed to inhibit cardiolipin synthesis also disable respiratory complex I functions [70, 71], which have been recently reported to sustain NLRP3 activation [72]. Thus, it is possible that downregulation of the enzyme cardiolipin synthase (CLS) could impair NLRP3 inflammasome [25] also by partially interfering with normal complex I activity. Intriguingly, treatment with the saturated fatty acid palmitate, which has been suggested to limit NLRP3 localization to mitochondria by diminishing the content of cardiolipin [25], also reduces the interaction between ER and mitochondria [73, 74]. Based on these findings, it might be assumed that cardiolipin, in concert with ER-mitochondria association, serves as regulator for optimal NLRP3 activation by driving the assembly of the inflammasome complex to the mitochondrial surface.

In conclusion, we identified the ER-mitochondria association as a crucial factor for NLRP3 activation and revealed a new mechanism in the regulation of the wound healing process that might be clinically relevant. For example, minimizing the adverse effects of LZD, using combined therapies, could be extremely significant, especially for the treatment of complicated skin and skin-structures infections that require prolonged use of LZD, including diabetic foot ulcers or chronic wounds.

## Materials and methods

### Bacterial strain and in vitro *S. aureus* stimulation

*S. aureus* ATCC 43300 (methicillin-resistant and *mecA*-positive strain) was the strain used for all the experiments. A *S. aureus* culture was established in 3 ml of Brain Heart Infusion broth (BHIB) until reaching an OD_650nm_ of ~0.4 to achieve the log phase of growth. Then, *S. aureus* was diluted in medium without antibiotics and added to HaCaT or nHEK cells with a MOI of 50 and 5, respectively. After 2 h of incubation, cells were not washed and treated either with vehicle (water), vancomycin (10 mg/L), or LZD (25 mg/L) for 4 h. For experiments with MRSA-S, *Staphylococcus* supernatant was recovered from an O/N broth culture. Briefly, *S. aureus* ATCC4300 was inoculated in 10 ml of BHIB O/N at 37 °C. The culture was centrifuged at 8000 g for 10 min. Pellet was discharged and the supernatant was recovered by filtration with a 0.22 µm syringe filter.

### Animals

In vivo studies were conducted with the approval and oversight of the Institutional Animal Care Committee of the Ministry of Health and by the Animal Research Ethics Committee of IRCCS INRCA (Istituto di Ricovero e Cura a Carattere Scientifico–Istituto Nazionale di Riposo e Cura per Anziani) (authorization 767/2016 Pr 28/7/2016). Appropriate sample size was calculated by PS software (biostat.mc.vanderbilt.edu/twiki/bin/view/Main/PowerSampleSize).

Male mice (3–6 months) weighting 28–32 g were used in the wound infection model. Animals were housed in individual cages following local and European guidelines, with constant temperature (22 °C), humidity, 12-h light/dark cycle, and food and water “*ad libitum*”.

### Wound infection model

The wound infection model was established as previously described [23]. Briefly, the study included a total of 36 animals, divided into 4 groups. *S. aureus* ATCC43300 was inoculated O/N at 37 °C in BHIB and diluted in saline to a final concentration of 5 × 10^7^ CFU/ml.

Mice were anesthetized by an intramuscular injection of ketamine (50 mg/kg of body weight) and xylazine (8 mg/kg of body weight), the hair on their back was shaved, and the skin was cleansed with 10% povidone-iodine solution (no animals dropped out due to infection or anesthetics). One full-thickness wound for each animal was established through the panniculus carnosus on the back subcutaneous tissue of each animal. A small piece of gauze was placed over each wound and then inoculated with 200 µl of bacterial culture or sterile saline in control group. The pocket was closed using skin clips. This procedure resulted in a local abscess at 24 h. The animals were returned to individual cages and thoroughly examined daily. After 24 h, the wound was opened and washed with saline, the gauze was removed, and treatment started. Mice were treated with daily intraperitoneal administration of 200 µl of saline or intraperitoneal vancomycin or LZD (10 mg/kg). For NLRP3 inflammasome inhibition, mice were treated with MCC950 (20 mg/kg).

After 10 days, animals were euthanized, and a 1 × 2-cm area of skin that included the wound was excised. To determine the bacterial burden, samples were homogenized using a stomacher with 1 ml of PBS. The suspensions were serially diluted in saline and dilution-containing bacteria were plated in mannitol-salt agar plates. After 24–48 h of incubation at 37 °C, viable bacteria were counted (detection limit 100 CFU/ml).

### Histological and immunohistochemical staining

The wound biopsies, including the epidermis, the dermis, and the subcutaneous panniculus carnosus muscle, were surgically removed at the time of euthanasia, fixed with 10% neutral buffered formalin and processed according to the standard routine light microscope tissue protocols. The processed tissues were embedded in paraffin, and serial sections (5.0 µm thick) were mounted on glass slides, and stained separately with hematoxylin and eosin (H&E), picrosirius red (365548 Sigma-Aldrich, Merck Life Science S.r.l., Milan, Italy) and immunohistochemical staining.

For all assays, digital images were obtained using a Nikon Eclipse E600 microscope and analyzed using NIS Elements image analysis software (Nikon Instruments, EuropeBV, Kingston, Surrey, England). The tissue sections by two independent investigators without knowledge of the previous treatment were scored.

The H&E stained sections were observed and scored according to a 5-tiered wound repair grading system (Supplementary Table 1). The quantitative measure of Sirius Red-stained collagen fibers was calculated as the percentage of positive collagen content by digital image analysis (ImageJ software) obtained using both brightfield and fluorescent microscopy. Each image was converted to 8-bit and then to 16-colors Lookup Table (LUT). After assigning a threshold, the dermis was selected as the region of interest (ROI), and the red staining percentage (collagen) area was evaluated.

From the paraffin-embedded tissues, some sections were prepared for immunohistochemical staining according to [75] and incubated with the antibodies reported in Supplementary Table 4. Two investigators (GL and CL), who were blind to the mouse outcome, evaluated separately the reactivity for the antibodies in at least 10 fields/sample (area of field 0.07 mm^2^) randomly selected at ×20 magnification. The number of positive stained cells was counted and estimated as the percentage of the total cells counted. The *k* value was >0.80, showing a substantial agreement between the two observers and among different observations of the same observer. Cells showing membranous or cytoplasmic staining were considered positive.

### Monitoring MRSA infection in live using a 3D holotomographic approach

HaCaT were seeded in 35 mm dishes (Ibidi GmbH, Gräfelfing, Germany) in medium without antibiotics. The day after, cells were infected with MRSA (MOI of 50) and monitored over time using a 3D Cell Explorer-fluo microscope (Nanolive, Ecublens, Switzerland), equipped with a 60× objective. Refractive index (RI) images (holographic reconstructions) were acquired every 2 min for 6–8 h and processed using a custom-made macro (based on “max projection”) in the Fiji software.

### Cell culture and transfection

Human immortalized keratinocytes (HaCaT), normal human dermal fibroblasts (NhDF), and Human Embryonic Kidney cells (HEK293FT) were cultured in High Glucose Dulbecco’s Modified Eagle Medium (HG-DMEM; Corning Inc., Corning, NY, USA), supplemented with 10% fetal bovine serum (Corning Inc.), 1% L-glutamine (Thermo Fisher Scientific, Waltham, MA, USA) and 1% penicillin/streptomycin (Thermo Fisher Scientific). Normal Human Epidermal Keratinocytes (nHEK) were cultured in Keratinocyte-SFM 1X (Life Technologies, Carlsbad, CA, USA), supplemented with 30 μg/ml Bovine Pituitary Extract (BPE), 0.2 ng/ml recombinant Epidermal Growth Factor (rEGF), and 5 mg/ml Gentamycin (Thermo Fisher Scientific). Cells were incubated at 37 °C with 5% CO2. All the cell lines were tested to prove the absence of mycoplasmas.

For each experiment, HaCaT cells were seeded at a cell density of 5 × 10^4^ cells/cm^2^, and nHEK cells were seeded at a cell density of 3.5 × 10^3^ cells/cm^2^.

For transient PDZD8 and GSDMD silencing, Stealth™ siRNA for PDZD8 (Invitrogen, Carlsbad, CA, USA) and pre-designed GSDMD siRNA (Sigma-Aldrich) were complexed with Lipofectamine RNAiMAX (Invitrogen) by reverse transfection (final concentration 40 nM), according to the manufacturer’s instruction. Stealth™ RNAi negative control Medium CG Duplex (Invitrogen) was used as negative control. Cells were transfected twice (the first at time 0, the second after 36 h) for 72 h overall, before processing cells.

Transient transfections were performed using polyethylenimine (PEI) (#23966, Polysciences, Warrington, PA, USA) as transfection reagent, with a DNA:PEI ratio of 1:6 for 16 h.

For stable PDZD8 silencing, lentiviral particles for shPDZD8 or empty pLKO were produced in HEK293FT cells as previously described [76]. The collected supernatant containing lentiviral particles was used to transfect HaCaT. Transfected clones were selected by adding 2 µg/ml puromycin to the culture medium.

For experiments with NLRP3 Inhibitor, MCC950 (Sigma-Aldrich) was diluted into the medium at the final concentration of 10 μM. For experiments with Interleukin-1 Receptor Antagonist, Anakinra (MedChemExpress, Monmouth Junction, NJ, USA) was used at the final concentration of 5 μg/ml.

### SDS-PAGE and immunoblotting

Cells were seeded in 6-well plates, treated as indicated above, and then lysed in Denaturing Lysis Buffer (50 mM Tris-HCl, 150 mM NaCl, 1% Triton X-100, 0.1% Sodium Dodecyl Sulfate), supplemented with 1 mM PMSF, protease inhibitors (Sigma-Aldrich) and PhosStop (Roche, Basil, Switzerland). The supernatants were collected after centrifugation at 12,000 × *g* for 10 min at 4 °C. Total protein amount was measured by DC protein assay (Bio-Rad, Hercules, CA, USA). Samples (10 µg of protein for each sample) were prepared using NuPAGE™ LDS Sample Buffer 4× (Invitrogen) and NuPAGE™ Sample Reducing Agent 10X (Invitrogen) and fractionated in Bolt™ 4 to 12%, Bis-Tris, 1.0 mm, Mini Protein Gels (Invitrogen).

Proteins were electrophoretically transferred to 0.2 µm nitrocellulose membranes (Bio-Rad). Membranes were incubated with 5% milk in Tris-Buffered Saline containing 0.1% Tween 20 (TBS-T) to block non-specific sites and then with primary antibodies at 4 °C overnight (Supplementary Table 4).

After three washes with TBS-T for 15 min, membranes were incubated with the appropriate secondary antibodies. Membranes were treated with Clarity Western ECL Substrate (Bio-Rad), and images were acquired with Alliance Mini HD9 (Uvitec, Cambridge, UK). Densitometric analysis was performed with Fiji software. (https://imagej.net/software/fiji/downloads) Images of original western blots are available as Supplementary Materials.

To evaluate secreted Caspase-1, cell supernatants were collected and 1 volume of 20% TCA was added to 1 volume of supernatant before incubation at −20 °C for 1 h. The solutions were centrifuged at 15,000 × *g* for 15 min at 4 °C, the supernatants were removed, and the pellets were resuspended in ice-cold acetone and incubated at −20 °C for 1 h, vortexing every 20 min. After centrifugation at 13,000 × *g* for 15 min, the supernatants were discarded, and the pellets were air-dried before re-suspending in Denaturing Lysis Buffer.

### 2D wound healing

HaCaT were seeded in a 12-well plate, in duplicate. After reaching 100% confluence, cells were treated as stated above and a wound was performed in each well by a 200 µl tip. Images were taken at time point 0 and 24 h after wound execution. The covered area, expressed as percentage, was analyzed by Fiji software.

### ELISA

To quantify secreted IL-1β, cells were seeded in 48-well plates and treated as mentioned above. The supernatant was collected and stored at −80 °C until use. ELISA assay was executed with Quantikine® ELISA Human IL-1β/IL-1F2 Immunoassay (DLB50, R&D Systems, Minneapolis, MN, USA) for HaCaT and Human IL-1β ELISA Kit (KHC0011, Invitrogen) for nHEK, according to manufacturer’s instruction. Absorbance was read at 450 nm using MultiskanGO plate reader (Thermo Fisher Scientific).

### Immunofluorescence

Cells were seeded on 13 mm diameter coverslips and treated as indicated, before fixing with 4% paraformaldehyde in PBS for 10 min at RT and then washing with PBS. Permeabilization was performed in 0.1% Triton X100 in PBS (PBS-Triton) and non-specific antigen blocking was conducted in 5% milk in PBS-Triton. Primary antibodies (dilution 1:100) (Supplementary Table 4) were incubated at 4 °C overnight. 488- and 594-labeled secondary antibodies (dilution 1:1000) were used for antigen visualization, before mounting with ProLong™ Diamond Antifade Mountant with DAPI (Invitrogen). Images were acquired with a confocal microscope (Zeiss LSM510) using a 63 × 1.4 NA Plan‐Apochromat oil‐immersion objective.

The fraction of NLRP3 overlapping mitochondria has been calculated using the JACoP plugin, available in Fiji.

For the analysis of mitochondrial morphology, morphometry parameters, and network connectivity were calculated using the plugin “Mitochondria Analyzer” [77], available in Fiji.

### FLICA-Caspase1 assay

FAM-FLICA kit (ICT098, Bio-Rad), was used for detecting caspase1 activity. For microscopic analysis, HaCaT cells were seeded in 24-well plates and treated as indicated. Cells were then labeled with FAM-FLICA, covered with ProLong™ Diamond Antifade Mountant with DAPI (Invitrogen), and observed with a fluorescent microscope Eclipse 600 (Nikon). NIS-Elements microscope imaging software (Nikon) was used to take images. Parallel experiments have been performed for quantification of fluorescence. HaCaT cells were seeded in 24-well plates, treated as indicated, trypsinized to create suspension, and then labeled with FAM-FLICA kit, according to the manual’s instructions. In the end, cells in suspension were aliquoted into a black 96-well plate and the fluorescent signal was detected by fluorescence microplate reader Infinite 200 PRO (Tecan, Männedorf, Switzerland) with excitation/emission at 485 nm/535 nm.

### XTT assay

HaCaT were seeded in 96-well plates and infected as already described. Cell viability was evaluated at different time points (0, 3, 6, 16, and 24 h) by sodium 3′ -[1- (phenylaminocarbonyl)- 3,4- tetrazolium]-bis (4-methoxy6-nitro) benzene sulfonic acid hydrate (XTT) colorimetric assay (Roche), according to the manufacturer’s instruction. Absorbance at 555 nm was read with 655 nm as reference wavelength using MultiskanGO plate reader (Thermo Fisher Scientific).

### Isolation of cytosolic and mitochondrial fractions

To separate mitochondrial and cytosolic proteins, cell fractionation was performed as indicated by [76]. Briefly, cells grown on 100 mm dishes were washed with cold PBS, scraped in ice, resuspended in Homogenization Buffer pH 7.4 (250 mM Sucrose, 50 mM Tris-HCl, and 2 mM EGTA) and homogenized in 1 ml glass Wheaton Potter in ice. A small part of the homogenate (100 µl) was centrifuged at 16500 g for 30’ at 4 °C and the supernatant was stored as the cytosolic fraction. The remaining part was centrifuged three times for 3 min at 600 × *g* at 4 °C to pellet and remove residual unbroken cells and nuclei. Then, the supernatant was centrifuged for 10 min at 10,000 × *g* at 4 °C to pellet the fraction containing crude mitochondria. The resulting pellet was lysed in Denaturing Lysis Buffer supplemented with PMSF, protease inhibitors, and PhosStop, then processed for SDS-PAGE and immunoblotting as described above.

### OCR and ECAR measurements (Seahorse)

The OCR and ECAR were measured using the real-time flux analyser XF-24e (Seahorse Bioscience). In brief, 6 × 10^4^ were infected and treated as indicated above. For OCR, cells were treated with 1 μM oligomycin, 2 μM carbonyl cyanide-p-trifluoromethoxyphenylhydrazone (FCCP), rotenone and antimycin A (both 1 μM) (all Sigma-Aldrich). For ECAR, cells were treated with 10 mM glucose, 1 μM oligomycin, and 50 mM 2-deoxyglucose (2-DG). Analysis was performed according to the XF Glycolysis Stress Test Kit User Guide. At the end of the run, cells were lysed using Denaturing Lysis Buffer. The total protein amount was measured by DC protein assay (Bio-Rad). OCR and ECAR were normalized to the total protein content as indicated.

### Mitochondrial superoxide measurements

Cells grown in 96-well plates were treated as indicated, washed two times with PBS, and stained for 30 min at 37 °C with 5 µM MitoSox (Thermo Fisher Scientific) diluted in HBSS supplemented with 5 mM glucose. Then, cells were washed three times with PBS and bathed with HBSS + glucose. Measurements were performed using the fluorescence microplate reader Infinite 200 PRO (Tecan). For each experiment, a positive control - consisting of cells treated with 50 µM Antimycin A (Sigma Aldrich) for 1 h – has been used as reference.

### DNA extraction and RT-PCR for cytosolic mtDNA (cmtDNA) evaluation

Cells were seeded in 6-well plates and treated as already described. Cells were washed with PBS, detached, and suspended in 1 ml PBS. A smaller amount of each cell suspension (200 µl) was used to obtain DNA and mtDNA from whole cells (WC) for cmtDNA normalization.

The remaining suspension (800 µl) was centrifuged and the obtained cell pellet was resuspended in an Extraction Solution (220 mM mannitol, 70 mM sucrose, 20 mM HEPES-KOH pH 7.5, 1 mM EDTA, 2 mg/ml BSA) containing protease inhibitors (Sigma-Aldrich) and PhosStop (Roche). The suspension was maintained in ice during homogenization with a 26 G needle syringe. A first centrifugation was performed at 1000 × *g* for 15 min at 4 °C to throw out the remaining whole cells, and a second centrifugation at 10,000 × *g* for 10 min at 4 °C was performed to save the supernatant with the cytosolic part. The purity (no mitochondrial contamination) of the cytosolic fractions has been assessed by western blotting. The WC and the cytosolic extracts were processed with QIAamp Mini Kit (Qiagen, Hilden, Germany) and QIAamp DNA Blood Mini Kit (Qiagen) respectively. RT-PCR was conducted on DNA from WC and cytosolic extracts using HOT FIREPol® EvaGreen® qPCR Supermix, 5× (Solis Biodyne, Tartu, Estonia). Primers were listed in Supplementary Table 5. cmtDNA relative expression (2^−ΔΔCq^) was obtained calculating Cq (Ct(WC mtDNA) – Ct(WC GAPDH), ΔCq (Ct(cmtDNA) – Cq), and ΔΔCq (ΔCq - ΔCq CTR).

### Extramitochondrial mtDNA evaluation through confocal microscopy

Cells were seeded in 35 mm dishes (Ibidi GmbH) and treated as described above. Cells were then incubated with PicoGreen™ dsDNA Assay (1:400, P7589, Invitrogen) and with 150 nM MitoTracker Red (M7512, Invitrogen). Cells were incubated with dyes for 30 min, washed 3 times with PBS, and then immediately observed. Images were taken with an inverted microscope Eclipse Ti2-E supplied with an AX confocal system (Nikon) at 60x magnification. Analysis was performed based on previous indications [78, 79]. Briefly, the red and green channels were separated and tresholded using the Otsu’s method. Then, the mitochondrial mask was subtracted from the DNA channel (PicoGreen dots/nucleoids) using the “Image calculator” available on Fiji, and the non-mitochondrial signals were identified using “Analyze particles”. The percentage of extramitochondrial puncta have been calculated on the total number of PicoGreen dots, resulted from the thresholded green channel (% of extramitochondrial dots x cell).

### ER-mitochondria contacts evaluation through confocal microscopy

Cells were seeded in 24 mm coverslips, transiently transfected for 16 h with GFP-SEC61B (Addgene, plasmid #121159) and mito-mCherry to label ER and mitochondria, respectively, and then treated as indicated. Images were acquired with a confocal microscope (Zeiss LSM510) using a 63 × 1.4 NA Plan‐Apochromat oil‐immersion objective. Co-localization analysis has been performed using the JACoP plugin, available in Fiji, for the calculation of M1 and M2 Manders’ coefficients.

### ER-mitochondria contacts evaluation through BRET technique

Analysis of ER-mitochondria contacts using BRET has been performed as previously described [51], with slight modifications. Briefly, cells seeded in white 96-well plates were co-transfected with Donor (RLuc8-L1, targeted to the mitochondria) and Acceptor (mVenus-L1, targeted in the ER) in a 1:3 ratio. Transfection of RLuc alone served as background luminescence, whereas the fusion protein RlucL1-mVenus was used as positive BRET control (maximum BRET values). After 16 h of expression, cells were treated as indicated and then washed 2 times in PBS. Freshly prepared 5 µM coelenterazine h (#C6780, Invitrogen) was added to the cells and incubated for 5 min in the dark. Double-color luminescence was immediately read with a microplate reader Infinite 200 PRO (Tecan). BRET signal was measured as milli BRET Unit (mBU), obtained by the calculation of acceptor/donor emission ratio and corrected by subtraction of the BRET value derived from RLuc alone.

### Complex IV activity

HaCaT were seeded in 100 mm dishes and three different LZD concentrations were tested (5 µg/ml, 10 µg/ml, 25 µg/ml). Cells were collected and lysates were used to determine Cytochrome C oxidation by Complex IV Human Enzyme Activity Microplate Assay kit (#ab109909, Abcam, Cambridge, UK), following the manufacturer’s protocol. Measurements were taken in a kinetic mode, reading absorbance at 550 nm every 5 min by MultiskanGO plate reader (Thermo Fisher Scientific) and the difference in the activity was measured using the 55th and 115th-minute reading.

### Mitochondrial membrane potential (∆Ψ) measurements

Cells were seeded on 35 mm dishes (Ibidi) and treated as described. Mitochondrial ∆Ψ was measured by loading cells with 10 nM tetramethyl rhodamine methyl ester (TMRM, Invitrogen) for 30 min at 37 °C. Successively, cells were imaged over time using a 3D Cell Explorer-fluo microscope (Nanolive, Switzerland). Basal levels were normalized on fluorescence in the presence of 10 μM FCCP.

### Mitochondrial Ca^2+^ uptake analysis

Cells were seeded on 13 mm coverslips, transiently transfected with mutated mitochondrial matrix-targeted aequorin (mtAEQmut) for 16 h, and treated as indicated. Then, cells were incubated with 5 μM coelenterazine for 1 h and allocated in a thermostated (37 °C) chamber located in close proximity to a low-noise photomultiplier with a built-in amplifier/discriminator for luminescence detection. Cells were perfused with Krebs–Ringer modified buffer (KRB: 125 mM NaCl, 5 mM KCl, 1 mM Na_3_PO_4_, 1 mM MgSO_4_, 5.5 mM glucose, and 20 mM 4-(2-hydroxyethyl)-1-piperazineethanesulfonic acid [HEPES], pH 7.4, at 37 °C) supplemented with 1 mM CaCl_2_ and Ca^2+^ rise was evoked by stimulation with 100 µM ATP. Cells were then lysed with Triton X-100 in a hypotonic Ca^2+^-rich solution (10 mM CaCl_2_ in H_2_O), thus discharging the remaining aequorin pool. The aequorin luminescence data were collected and calibrated into [Ca^2+^] values using the appropriate algorithm reported in [80].

### RT-PCR

Total RNA was extracted from cells treated for both transient and stable transfection using Quick-RNA™ Miniprep Kit (Zymo Research, Irvine, CA, USA) and stored at -80 °C until use. Retrotranscription reaction was performed with All-In-One 5X RT MasterMix (Applied Biological Materials Inc., Richmond, Canada). RT-PCR was performed to amplify the obtained cDNA using HOT FIREPol® EvaGreen® qPCR Supermix, 5x (Solis Biodyne, Tartu, Estonia). PDZD8, GSDMD, and GAPDH primers were used (Supplementary Table 5). The mRNA expression was calculated using the ΔΔCt method and showed as Relative Expression.

### 3D-wound healing model

A 3 mg/ml Collagen solution was prepared with Cultrex® Rat Collagen I (#440-100-01, R&D Systems) and culture medium, and NaOH 1 N was used to adjust pH to 7. All ingredients were stored on ice before and during the preparation and mixed in the exact order listed above. 1×10^5^ NhDF/ml was added to the solution, and 1.5 ml collagen discs were prepared in 12-well plates. After 5 days, 5 × 10^4^ HaCaT/cm^2^ were seeded on the top of half collagen discs and cultured for 7 days, before practicing the wound with a 2-mm biopsy punch. Wounded discs were glued on the top of HaCaT-free discs with 100 µl collagen solution and wounds were filled with 20 µl collagen solution. Diluted (20%) MRSA-S was used for mimicking chronic inflammation and 50% of medium was replaced daily for 10 days. For experiments of Fig. 4h, i, LZD was added daily, starting from day 2 (in combination with MRSA-S) and then alone from day 3 to day 10. Wound models were fixed in 4% PFA in PBS for 1 h at 4 °C and washed with PBS. Immunofluorescence was performed as described above, using an anti-K10 antibody (Supplementary Table 4) overnight at 4 °C. Images were acquired with an inverted microscope Eclipse Ti2-E supplied with an AX confocal system (Nikon) at ×4 magnification. Wound closure was calculated by Fiji software. Briefly, single images were obtained by z-projection of z-stack acquisition (Sum Slices projection), an arbitrary threshold was set to discriminate fluorescence from the background and the wound closure was measured.

### Statistical analysis

Errors bars in graphs represent the mean ± SD from at least three independent experiments. Statistical significance was analyzed using a two-tailed, unpaired, Student’s *t*-test, or one-way or two-way ANOVA. All statistical analyses were performed using the GraphPad Prism software.

### Supplementary information

Supplementary information

Movie S1

Original western blots

## Data Availability

All data needed to evaluate the conclusions in the paper are present in the paper and the supplementary materials.
