# Supplementary material for: ER-mitochondria association negatively affects wound healing by regulating NLRP3 activation
Source: Cell Death Dis. 2024 Jun 11;15(6):407. doi: 10.1038/s41419-024-06765-9 (PMC11167056; doi:10.1038/s41419-024-06765-9)
Supplement: Supplementary file 1 — Supplementary information [file 41419_2024_6765_MOESM1_ESM.pdf]

Supplemental Information

ER-mitochondria association negatively affects wound healing by regulating NLRP3 activation.

Caterina Licini[1,], Gianluca Morroni[2,*], Guendalina Lucarini[1,*], Veronica Angela Maria Vitto[3], Fiorenza Orlando[4], Sonia Missiroli[3], Gloria D'Achille[2], Mariasole Perrone[3], Tatiana Spadoni[5], Laura Graciotti[5], Giorgia Bigossi[6], Mauro Provinciali[6], Annamaria Offidani[7], Monica Mattioli-Belmonte[1,6], Oscar Cirioni[8], Paolo Pinton[3], Oriana Simonetti[7,†], and Saverio Marchi[1,6,†].*

Corresponding authors: Saverio Marchi, and Oriana Simonetti

Email: s.marchi@univpm.it; o.simonetti@staff.univpm.it

This file includes:

Supplementary Figure 1 to 6

Supplementary Figure legends 1 to 6

Tables S1 to S5

Legends for Movie S1

Supplementary Figure 1

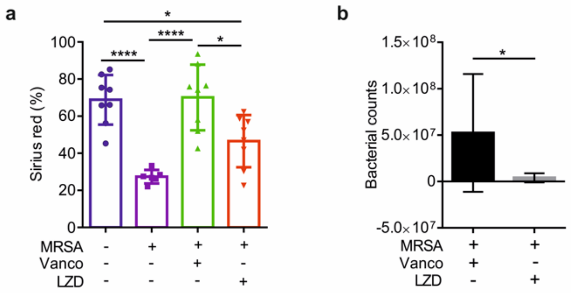

Supplementary Figure 2

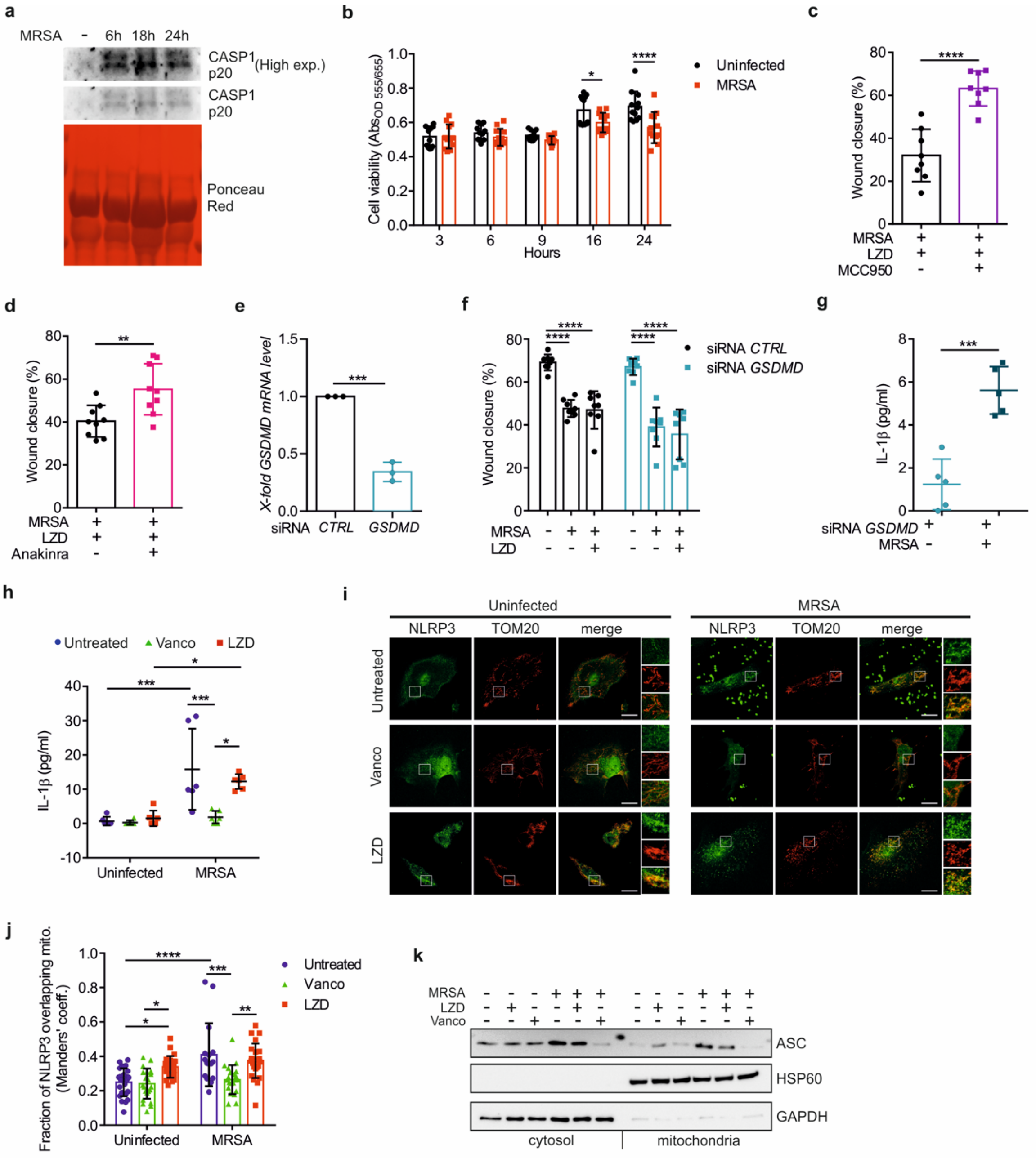

Supplementary Figure 3

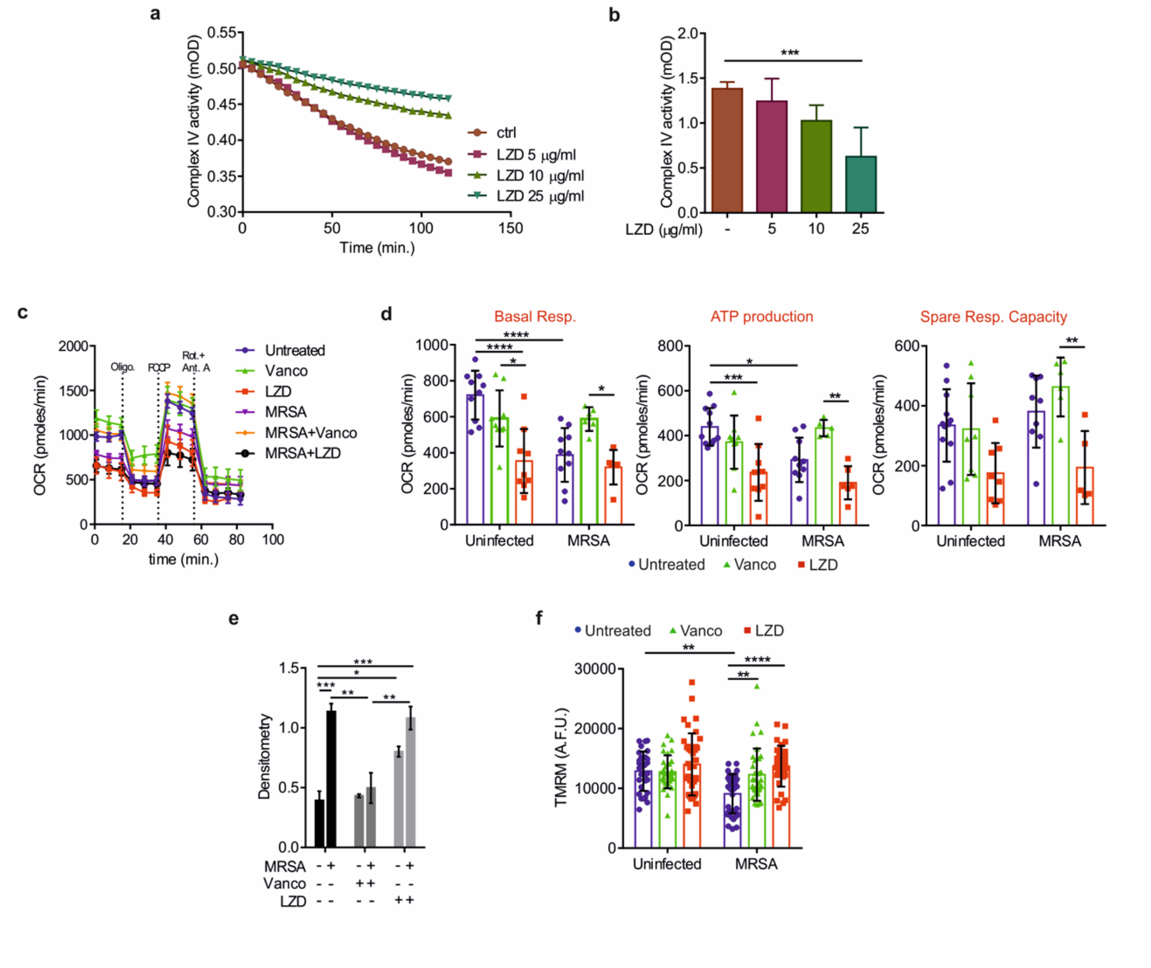

Supplementary Figure 4

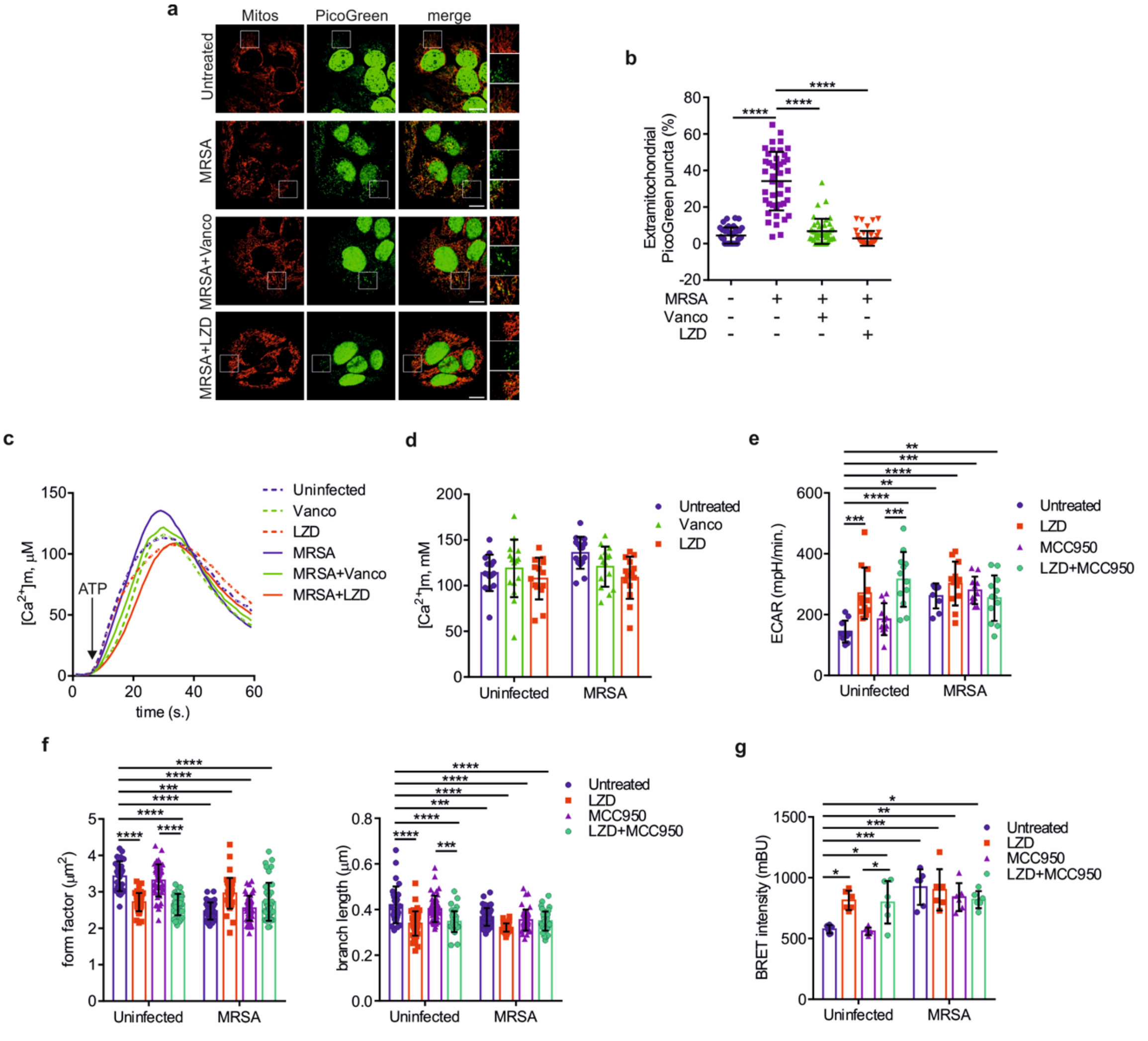

Supplementary Figure 5

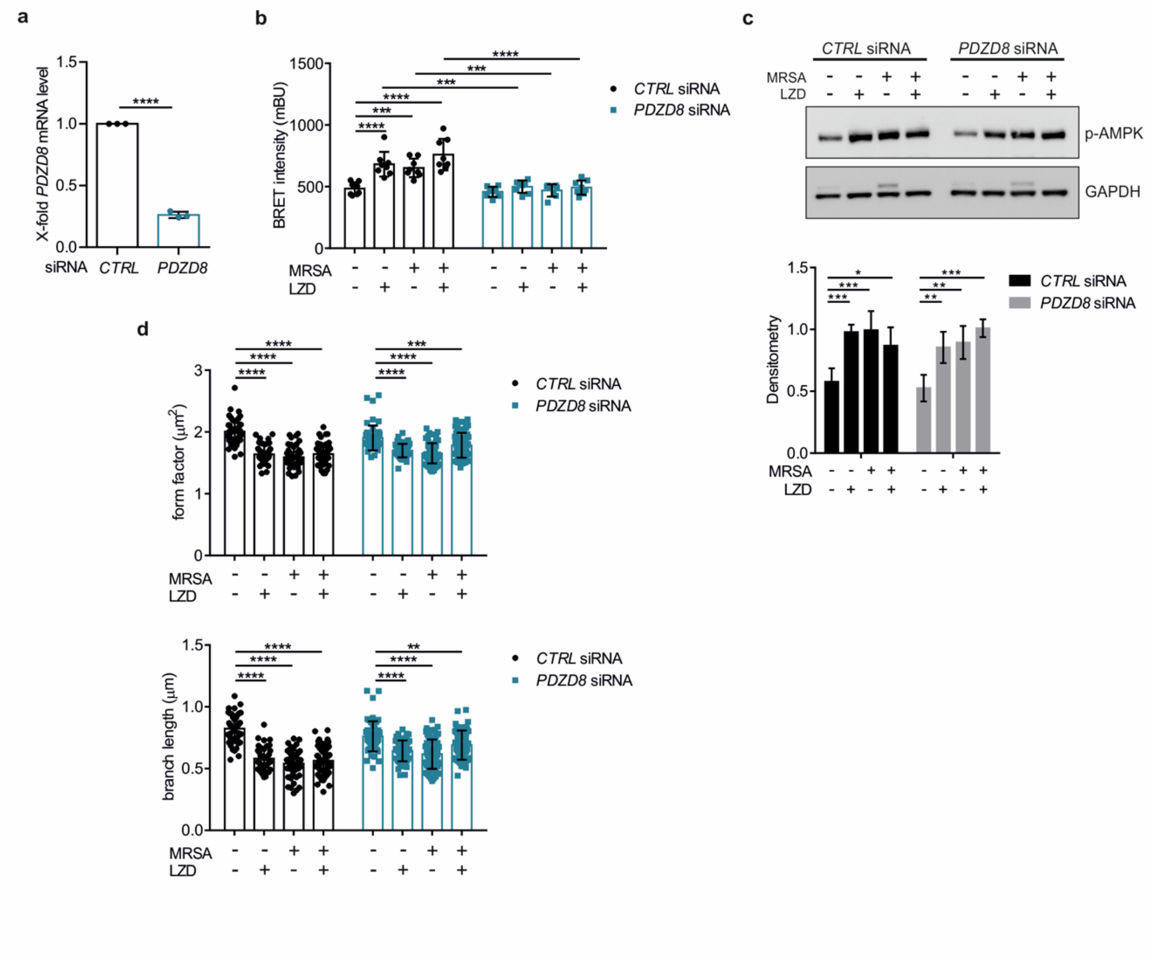

Supplementary Figure 6

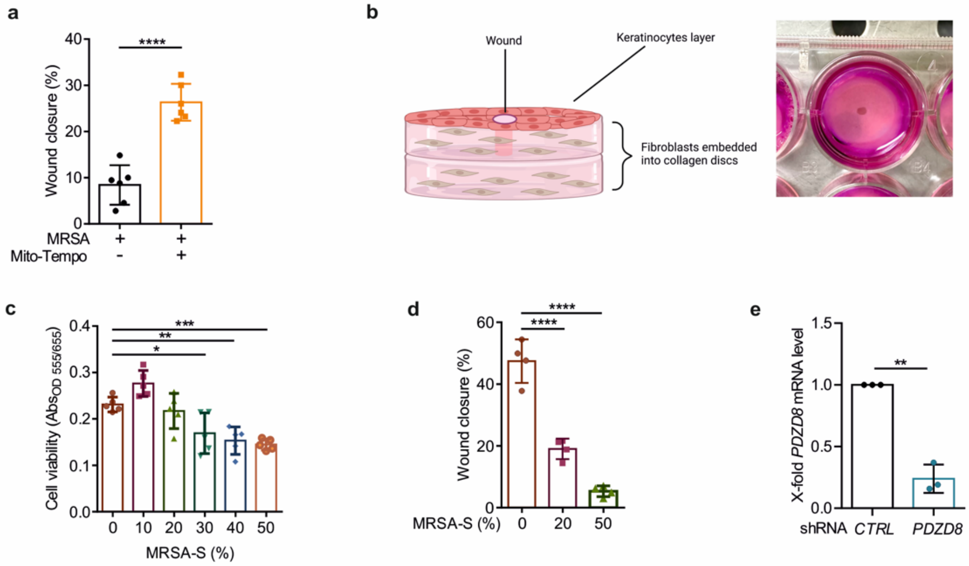

Supplementary Figure Legends

Supplementary Figure 1: Assessment of wound repair in a mouse model of MRSA-infected wound

a) Quantification of Sirius Red staining (showed as percentage), related to Fig. 1d (One-way ANOVA: * P=0.0159 [uninfected/untreated vs MRSA/LZD]; * P=0.0104 [MRSA/Vanco vs MRSA/LZD]; **** P<0.0001.
b) Quantification of bacterial burden in skin mice, treated as indicated (Student's T-test: * P=0.0369).

Supplementary Figure 2: MRSA and LZD induce NLRP3 association and activation to the mitochondrial compartment

a) Representative immunoblot for secreted Caspase 1 p20 into supernatants of HaCaT cells collected at 6, 18, and 24 h after infection with MRSA.
b) Cell viability observed by XTT assay in HaCaT at 3, 6, 9, 16, and 24 h after MRSA infection (and relative uninfected control). (n=3 independent experiments; Two-way ANOVA: * P=0.0309; **** P<0.0001)
c) Quantification of wound closure (showed as percentage) by LZD-treated/MRSA-infected HaCaT cells, with or without treatment with the NLRP3 inhibitor MCC950, 24 h after wound opening (n=4 independent experiments; Student's T-test: **** P<0.0001).
d) Quantification of wound closure (showed as percentage) by LZD-treated/MRSA-infected HaCaT cells, with or without treatment with the IL-1 receptor antagonist Anakinra, 24 h after wound opening (n=3 independent experiments; Student's T-test: ** P<0.0058).
e) GSDMD mRNA levels in control (negative siRNA) and *GSDMD*-silenced HaCaT cells (Student's T-test: *** P=0.0002).

f) Quantification of wound closure (showed as percentage) in control (negative siRNA) and *GSDMD*-silenced HaCaT cells, treated as indicated, 24 h after wound opening (n=4 independent experiments; Two-way ANOVA: **** P<0.0001).
g) Quantification of IL-1β secreted into supernatants from *GSDMD*-silenced HaCaT cells, infected or not with MRSA for 24 h, performed by ELISA assay (n=2 independent experiments; Student's T-test: *** P<0.0003).
h) Quantification of IL-1β secreted into supernatants from nHEK cells, treated as indicated, performed by ELISA assay (n=3 independent experiments; Two-way ANOVA: * P=0.0111 [uninfected/LZD vs MRSA/LZD]; * P=0.0149 [MRSA/Vanco vs MRSA/LZD]; *** P=0.0002 [uninfected/untreated vs MRSA/untreated]; *** P=0.0006 [MRSA/untreated vs MRSA/Vanco]).
i) Representative confocal immunofluorescence staining images of NLRP3 (green) and TOM20 (used as a mitochondrial marker, red) in nHEK cells, treated as indicated (scale bars: 5 μm). Merged images are shown. Magnification of TOM20, NLRP3, and merged images in insets.
j) Quantification of NLRP3/mitochondria association in nHEK cells, treated as indicated, by Manders' coefficient calculation (n=3 independent experiments; Two-way ANOVA: * P=0.0220 [uninfected/untreated vs uninfected/LZD]; * P=0.0121 [uninfected/Vanco vs uninfected/LZD] ** P=0.0041; *** P=0.0004; **** P<0.0001).
k) Mitochondrial and cytosolic fractions of HaCaT cells, treated as indicated, were analyzed by immunoblot. GAPDH and HSP60 have been used as markers for cytosol and mitochondria, respectively.

Supplementary Figure 3: Role of LZD on mitochondrial activity

a) Rate of Complex IV activity in HaCaT cells treated with different LZD concentrations, measured for 120 min.
b) Analysis of Complex IV activity, calculated between the 55th and 115th-minute reading, in HaCaT cells treated with different LZD concentrations (n=3 independent experiments; One-way ANOVA: *** P=0.0002).

c) Mitochondrial respiration measured using Seahorse in HaCaT cells, treated as indicated. n=3 independent experiments.

d) Quantification of basal respiration, ATP production, and spare respiratory capacity measured in HaCaT cells, treated as indicated (n=3 independent experiments; Two-way ANOVA: Basal resp.: * P=0.0113 [uninfected/Vanco vs uninfected/LZD]; * P=0.0234 [MRSA/Vanco vs MRSA/LZD]; **** P<0.0001. ATP prod.: * P=0.0168; ** P=0.0025; *** P=0.0002. Spare resp. capacity: ** P=0.0086)

e) Quantification of phosphorylated AMPK (p-AMPK) levels, referred to Fig. 3d. (n=3 independent experiments; Two-way ANOVA: * P=0.0318; ** P=0.0011 [MRSA/untreated vs MRSA/Vanco]; ** P=0.0024 [MRSA/Vanco vs MRSA/LZD]; *** P=0.0003 [uninfected/untreated vs MRSA/untreated]; *** P=0.0006 [uninfected/untreated vs MRSA/LZD]).

f) Analysis of mitochondrial membrane potential by TMRM probe in HaCaT cells, treated as indicated (n=3 independent experiments; Two-way ANOVA: ** P=0.0012 [uninfected/untreated vs MRSA/untreated]; ** P=0.0080 [MRSA/untreated vs MRSA/Vanco]; **** P<0.0001).

Supplementary Figure 4: LZD-dependent NLRP3 activation is a consequence of mitochondrial damage and it is not related to mtDNA release or mitochondrial Ca^{2+} entry

a) Representative confocal immunofluorescence staining images of mtDNA puncta (green) and mitochondria (red) in HaCaT cells, infected or not with the MRSA supernatant and treated as indicated (scale bars: 10 μm). Merged images are shown. Magnification of mitochondria, mtDNA dots, and merged images in insets.

b) Quantification of extramitochondrial mtDNA in HaCaT cells, treated as indicated (referred to Supp. Fig. 4a); (n=3 independent experiments; One-way ANOVA: **** P<0.0001).

c) Representative kinetics of mitochondrial Ca^{2+} uptake in mitochondrially-targeted aequorin (mt-AEQ mut)-transfected HaCaT cells, treated as indicated. Where indicated, cells were stimulated with 100 μM ATP to evoke a Ca^{2+} response.

d) Quantification of Ca^{2+} levels in mitochondrially-targeted aequorin (mt-AEQ mut)-transfected HaCaT cells, treated as indicated (n=3 independent experiments; Two-way ANOVA).
e) Quantification of glycolysis in HaCaT cells, treated as indicated (n=3 independent experiments; Two-way ANOVA: ** P=0.0057 [uninfected/untreated vs MRSA/untreated]; ** P=0.0052 [uninfected/untreated vs MRSA/LZD+MCC950]; *** P=0.0007 [uninfected/untreated vs uninfected/LZD]; *** P=0.0002 [uninfected/MCC950 vs uninfected/ LZD+MCC950]; *** P=0.0003 [uninfected/untreated vs MRSA/ MCC950]; **** P<0.0001).
f) Morphometric analysis of the mitochondrial network in HaCaT cells, treated as indicated, by calculation of the parameters "form factor" and "branch length" (n=3 independent experiments; Two-way ANOVA: form factor: *** P=0.0004 [uninfected/untreated vs MRSA/LZD]; **** P<0.0001. Branch length: *** P=0.0002 [uninfected/untreated vs MRSA/untreated]; *** P=0.0005 [uninfected/MCC950 vs uninfected/LZD+MCC950]; **** P<0.0001).
g) Quantification of bioluminescence intensity detected by BRET assay in HaCaT cells, treated as indicated (n=3 independent experiments; Two-way ANOVA: * P=0.0206 [uninfected/untreated vs uninfected/LZD]; * P=0.0382 [uninfected/untreated vs uninfected/LZD+MCC950]; * P=0.0196 [uninfected/MCC950 vs uninfected/LZD+MCC950]; * P=0.0382 [uninfected/untreated vs MRSA/LZD+MCC950]; ** P=0.0069 [uninfected/untreated vs MRSA/MCC950]; *** P=0.0002 [uninfected/untreated vs MRSA/untreated]; *** P=0.0005 [uninfected/untreated vs MRSA/LZD].

Supplementary Figure 5: Inhibition of ER-mitochondria association by PDZD8 silencing does not affect mitochondrial damage induced by LZD

a) PDZD8 mRNA levels in control (negative siRNA) and *PDZD8*-silenced HaCaT cells (Student's T-test: **** P<0.0001).
b) Quantification of bioluminescence intensity detected by BRET assay in control (negative siRNA) and *PDZD8*-silenced HaCaT cells, treated as indicated (n=4

independent experiments; Two-way ANOVA: *** P=0.0009 [CTRL siRNA uninfected/untreated vs CTRL siRNA MRSA/untreated]; *** P=0.0002 [CTRL siRNA uninfected/LZD vs PDZD8 sirna uninfected/LZD]; *** P=0.0002 [CTRL siRNA MRSA/untreated vs PDZD8 sirna MRSA/untreated]; **** P<0.0001).

c) Representative immunoblot and quantification (below) of phosphorylated AMPK (p-AMPK; Thr172) in control (negative siRNA) and *PDZD8*-silenced HaCaT cells, treated as indicated, (n=6 independent experiments; Two-way ANOVA: * P=0.0107; ** P=0.0042 [PDZD8 sirna uninfected/untreated vs PDZD8 sirna uninfected/LZD]; ** P=0.0016 [PDZD8 sirna uninfected/untreated vs PDZD8 sirna MRSA/untreated] *** P=0.0007 [CTRL siRNA uninfected/untreated vs CTRL siRNA uninfected/LZD]; *** P=0.0005 [CTRL siRNA uninfected/untreated vs CTRL siRNA MRSA/untreated]; **** P<0.0001).
d) Morphometric analysis of the mitochondrial network in control (negative siRNA) and *PDZD8*-silenced HaCaT cells, treated as indicated, by calculation of the parameters "form factor" and "branch length" (n=3 independent experiments; Two-way ANOVA: ** P=0.0013; *** P=0.0005; **** P<0.0001)

Supplementary Figure 6: Downregulation of ER-mitochondria contact formation restores healing in a 3D wound model

a) Quantification of wound closure (showed as percentage) after 24 h in MRSA-infected HaCaT, with or without Mito-Tempo treatment (n=3 independent experiments; Student's T-test: **** P<0.0001).
b) Representative drawing and image for 3D-wound model.
c) Cell viability in HaCaT cells after treatments with different MRSA-S at different percentages (n=3 independent experiments; One-way ANOVA: * P=0.0139; ** P=0.0018; *** P=0.0006).
d) Quantification of wound closure (showed as percentage) in HaCaT treated with 20% or 50% MRSA-S, 24 h after wound opening (n=2 independent experiments; One-way ANOVA: **** P<0.0001).

PDZD8 mRNA levels in pLKO-transfected (CTRL) and *PDZD8* shRNA-transfected stable HaCaT cells (n=3 independent experiments; Student's T-test: ** P=0.0071).

Table S1. Five-tiered grading system to evaluate wound healing.

Scores	Wound re-epithelialization	Collagen deposition	Inflammatory infiltrate
0	Minimal keratinocyte migration	None	Absent
1	Single-layer epithelium with partial closure	Trace, irregular	Scant
2	Multilayer epithelium partially to complete covering wound	Modest, irregular	Moderate
3	Hypertrophic epithelium and partial stratum corneum	Good, regular	Severe
4	Complete and normal closure	Marked, dense and regular	_

Table S2. Immunohistochemical evaluation of VEGF, MMP2, MMP9 and K10.

		VEGF			MMP2			MMP9			K10	
		Epit	Dermis	Tot	Epit	Dermis	Tot	Epit	Dermis	Tot	Wound center (Suprabasal/upper focal)	Wound edges (Suprabasal/upper focal)
G1	Uninfected Untreated	26.39 ± 2.5	40.78 ± 3.49	31.97 ± 2.56	10.09 ±1.36	14.66 ± 0.57	13.24 ± 1.59	14.61 ± 3.18	20.10 ± 1.61	17.33 ± 1.45	69.63 ± 2.38	72.63 ± 2.52
G2	MRSA	12.81 ± 0.64	4.0 ± 0.43	8.15 ± 2.29	30.50 ± 6.00	20.83 ± 2.15	25.08 ± 1.77	30.64 ± 2.34	45.04 ± 2.91	39.28 ± 3.48	25.89 ± 5.42	70.90 ± 5.25
G3	MRSA + Vanco	31.06 ± 3.10	30.56 ± 2.59	30.96 ± 2.60	10.27 ± 0.51	15.16 ± 1.47	13.56 ± 1.72	10.77 ± 3.47	22.07 ± 1.96	16.36 ± 1.73	60.78 ± 2.43	70.30 ± 1.84

G4	MRSA + LZD	20.06 ± 3.85	10.44 ± 2.50	15.12 ± 2.90	25.18 ± 1.42	15.47 ± 2.77	18.50 ± 3.32	15.09 ± 2.56	20.03 ± 1.75	17.18 ± 2.22	35.52 ± 4.96	75.66 ± 6.17

Table S3. Immunohistochemical evaluation of IL-1β and CASP1

	IL-1β				CASP1			
	Epithelial	Dermis	Infiltrate	Total	Epithelial	Dermis	Infiltrate	Total
Uninfected Untreated	-	-	-	-	-	-	-	-
MRSA	39.51 ± 2.01	54.38 ± 2.53	10.70 ± 2.02	36.03 ± 6.09	39.61 ± 1.74	52.41 ± 10.15	17.15 ± 6.13	43.97 ± 8.13
MRSA + Vanco	-	-	-	-	-	-	-	-
MRSA + LZD	40.24 ± 1.99	30.97 ± 5.42	20.52 ± 2.26	30.47 ± 5.02	34.27 ± 4.16	10.30 ± 3.36	5.90 ± 1.54	19.78 ± 1.11

Table S4. List of the antibodies

	Dilution	Source
Primary antibodies		
Anti- ASC	1:1000 (WB)	n. cat. AG-25B-0006-C100, Adipogen, San Diego, CA, USA
Anti-AMPK	1:1000 (WB)	n. cat. #2532, Cell Signaling, Danvers, MA, USA
Anti-Caspase 1 p20	1:100 (IHC)<br>1:500 (WB)	n. cat. AG-20B-0042-C100, Adipogen, San Diego, CA, USA
Anti-Cytokeratin 10	1:250 (IF)	n. cat. MA5-13705, Invitrogen, Waltham, MA, USA
Anti-GAPDH	1:10000 (WB)	n. cat. 60004-1-Ig, Proteintech, Manchester, UK
Anti-HSP60	1:1000 (WB)	n. cat. sc-59567, Santa Cruz Biotechnologies, Santa Cruz, CA, USA
Anti-IL 1β	1:200 (IHC)	n. cat. #12242, Cell Signaling, Danvers, MA, USA
Anti-MMP2 (8B4)	1:100 (IHC)	n. cat. sc-13595, Santa Cruz Biotechnologies
Anti-MMP9 (2C3)	1:300 (IHC)	n. cat. sc-21733, Santa Cruz Biotechnologies
Anti-NLRP3	1:100 (IF)	n. cat. AG-20B-0014-C100, Adipogen, San Diego, CA, USA

Anti-Phospho-AMPKα (Thr172)	1:100 (IHC)<br>1:1000 (WB)	n. cat. #2535, Cell Signaling, Danvers, MA, USA
Anti-TOM20	1:100 (IF)	n. cat. HPA011562, Sigma-Aldrich, Merck Life Science S.r.l., Milan, Italy
Anti-VEGF (C-1)	1:200 (IHC)	n. cat. sc-7269, Santa Cruz Biotechnologies
Secondary antibodies		
Anti-mouse HRP-conjugated	1:15000 (WB)	n. cat. A90-116P, Bethyl laboratories, Waltham, MA, USA
Anti-rabbit HRP-conjugated	1:10000 (WB)	cat. n° A16104, Invitrogen
Anti-goat HRP-conjugated	1:5000 (WB)	cat. n° 31402, Invitrogen
Goat anti-Mouse FITC-conjugated	1:1000 (IF)	cat. n° A16079, Invitrogen
Goat anti-Rabbit Alexa Fluor™ Plus 555	1:2000 (IF)	cat. n° A32732, Invitrogen
Goat anti-Mouse Alexa Fluor™ 546	1:1000 (IF)	cat. n° A11030, Invitrogen

Table S5. List and sequences of the used primers

Gene	*Primer*	*Sequence*
**PDZD8**	Fw	5’-GAAGGCCGTCTTAAAGTTACGTTG
	Rv	5’-CTGCTTAACTCAAGTGTGCAATGA
**GSDMD**	Fw	5’-ATGAGGTGCCTCCACAACTTCC
	Rv	5’-CCAGTTCCTTGGAGATGGTCTC
**GAPDH**	Fw	5’-AGCCACATCGCTCAGACAC
	Rv	5’-GCCCAATACGACCAAATCC
**mtCO1**	Fw	5’-CAGGAGTAGGAGAGAGGGAGGTAAG
	Rv	5’-TACCCATCATAATCGGAGGCTTTGG
**mtD-Loop**	Fw	5’-GTCCCTTGACCACCATCCTC
	Rv	5’-GTAGCACTCTTGTGCGGGAT

Movie S1. MRSA-infected HaCaT cells during the first 6 hours from infection.
