# Supplementary figures and images for: ER-mitochondria association negatively affects wound healing by regulating NLRP3 activation
Source: Cell Death Dis. 2024 Jun 11;15(6):407. doi: 10.1038/s41419-024-06765-9 (PMC11167056; doi:10.1038/s41419-024-06765-9)

Fig. 2e

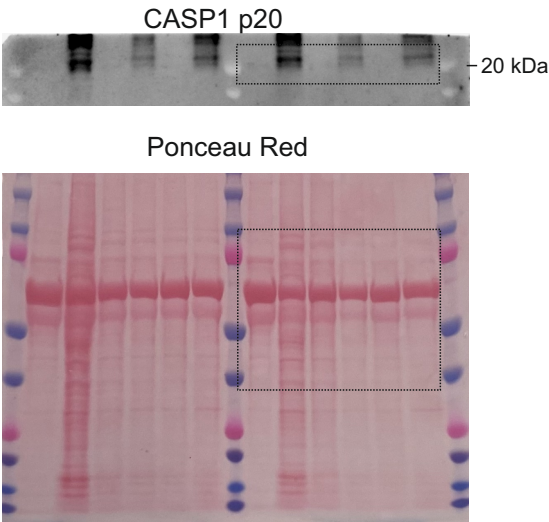

Fig. 3d

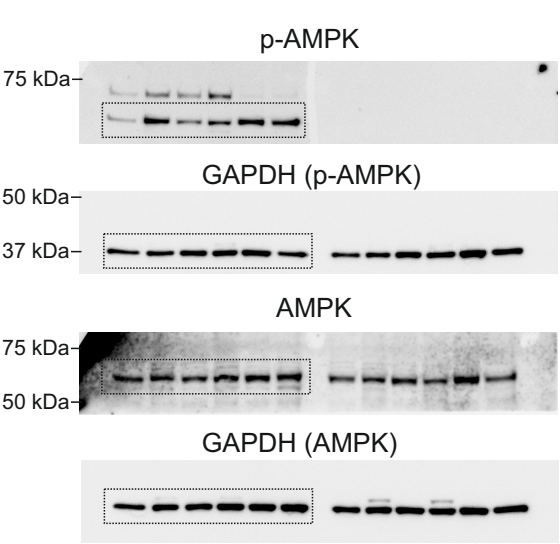

Supp. Fig. 2a

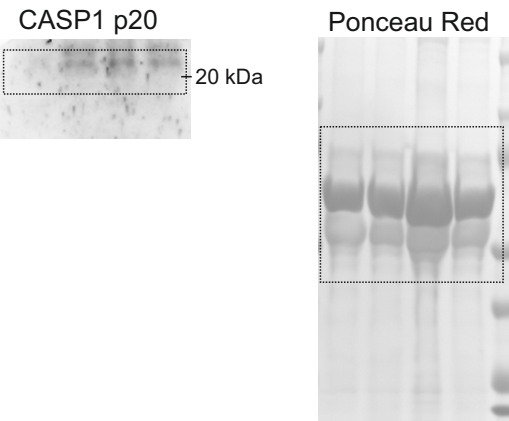

Supp. Fig. 2j

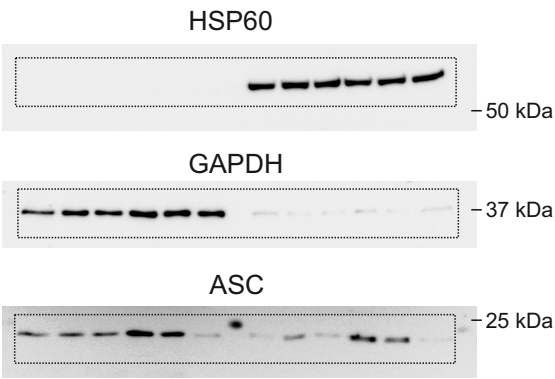

Supp. Fig. 5c

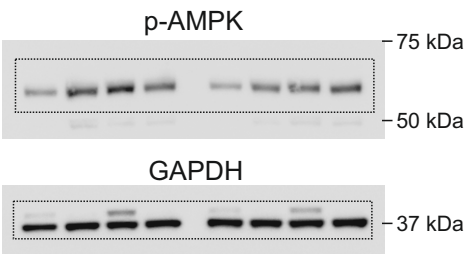

Supplement: Supplementary file 3 — Original western blots [file 41419_2024_6765_MOESM3_ESM.pdf]
